# Heuristics to Evaluate Interactive Systems for Children with Autism Spectrum Disorder (ASD)

**DOI:** 10.1371/journal.pone.0132187

**Published:** 2015-07-21

**Authors:** Kamran Khowaja, Siti Salwah Salim

**Affiliations:** Faculty of Computer Science and Information Technology, University of Malaya, 50603, Kuala Lumpur, Malaysia; Hamamatsu University School of Medicine, JAPAN

## Abstract

In this paper, we adapted and expanded a set of guidelines, also known as heuristics, to evaluate the usability of software to now be appropriate for software aimed at children with autism spectrum disorder (ASD). We started from the heuristics developed by Nielsen in 1990 and developed a modified set of 15 heuristics. The first 5 heuristics of this set are the same as those of the original Nielsen set, the next 5 heuristics are improved versions of Nielsen's, whereas the last 5 heuristics are new. We present two evaluation studies of our new heuristics. In the first, two groups compared Nielsen’s set with the modified set of heuristics, with each group evaluating two interactive systems. The Nielsen’s heuristics were assigned to the control group while the experimental group was given the modified set of heuristics, and a statistical analysis was conducted to determine the effectiveness of the modified set, the contribution of 5 new heuristics and the impact of 5 improved heuristics. The results show that the modified set is significantly more effective than the original, and we found a significant difference between the five improved heuristics and their corresponding heuristics in the original set. The five new heuristics are effective in problem identification using the modified set. The second study was conducted using a system which was developed to ascertain if the modified set was effective at identifying usability problems that could be fixed before the release of software. The post-study analysis revealed that the majority of the usability problems identified by the experts were fixed in the updated version of the system.

## Introduction

Autism is one of the five disorders under the umbrella of autism spectrum disorder (ASD), which includes autism, Asperger syndrome, childhood disintegrative disorder, Rett syndrome and pervasive development disorder-not otherwise specified (PDD-NOS). It is a neurological disorder characterised by impairment in social communication and restricted or repetitive behaviour, the symptoms of which appear during the first three years of a child’s life, and the impairment will take its toll during the later course of life. The behavioural changes caused by ASD may vary from child to child: one may be very verbal, bright and enthusiastic, while another may be non-verbal and intellectually challenged [1].

Innovation and advancement of information and communication technology have marked the start of the use of technology in the training and development programmes for children with ASD [2]. These children are visual learners and the use of human-computer interaction (HCI) principles has increased significantly in recent years in the design and development of interactive systems for those affected by ASD [3–5]. These days, children look beyond mere usable systems as they are attracted to systems that can provide a more interesting environment and are fun to use. Hence, evaluation needs to be carried out to assess not only the system usability but also user-friendliness and ease of use. There are two types of evaluations: the first is the summative evaluation, which is used to assess the success of systems once they are completed; and the second is the formative evaluation, which is performed during the design and development of systems to ensure they can meet user requirements.

The focus of formative evaluation is to identify and fix usability issues during the early stage of designing a system, and heuristic evaluation is one of the techniques that falls under the category of formative evaluation. It is an evaluation approach in which an expert applies the knowledge of typical end users and evaluates a system based on a set of recognised usability principles, so-called the ‘heuristics’, to find out the usability problems in the system [6]. This method of evaluation has gained popularity in the community of HCI since it was first introduced by Nielsen in the year 1990 [7], and the current trend is to develop and evaluate more specialised heuristics for new technologies and systems. Researchers have typically developed their own set of heuristics by modifying Nielsen’s heuristics together with design guidelines, market research, requirements documents of a specific product, expert reviews, researchers’ own experience in the area of research or a combination of these items [6]. There is a need to have specialised heuristics tailored to the interactive systems for children with ASD that can be used to identify and fix usability problems during the early stage of designing a system. However, based on the existing studies, we could not find any specialised heuristics that were applicable to systems for children with ASD.

In this study, we use the existing guidelines for interactive systems designed for children with ASD and adapt the original set of heuristics by Nielsen to create a modified set of heuristics for these systems. The structure of the rest of this paper is as follows: we first provide the details of related work in the areas of interactive systems for children with ASD and heuristic evaluation; secondly, we provide a description of the method used to develop the modified set of heuristics; thirdly, we present the two studies carried out along with their results indicating the effectiveness of the modified set, and finally the paper is concluded with discussions on the findings of this research.

## Related Work

### 2.1. Interactive systems for children with ASD

The challenge of caregivers of children with ASD is helping them to attain the goal of living a happy and satisfying life [8]. Education plays a vital role in achieving this goal as “there is no known cure for autism … it is important to view autism as one form of mental disability that will require special teaching and guidance rather than a psychiatric illness requiring therapy” [9].

Interactive systems for children with ASD are defined as systems that respond in real-time and in a personalised manner by understanding the behaviours of each child [4]. This is a software-based solution that engages the interest of children in a comprehensively designed system focusing on specific behaviours. At present, the applications of interactive systems have been proven to change the lives of children with ASD [10,11]. The outcomes evidently show that the employment of these systems helps the affected children to interact, socialise, communicate and learn in novel ways. Various reviews [12–17] have been carried out on teaching different skills to children with ASD by using interactive systems. Nearly all the studies in the above-mentioned reviews demonstrated improvement in the learning of these children after they had used the interactive systems. In this paper, our emphasis is to create a specialised set of heuristics to meet the purpose of formative evaluation of interactive systems for children with ASD so that an engaging system can be developed.

### 2.2. Heuristic evaluation

Jakob Nielsen in collaboration with Rolf Molich, developed a set of 10 usability principles called heuristics to evaluate if elements present in a user interface, for instance menu, dialogue boxes, help, etc., follow the principles [18]. He named them heuristics because they resembled the rule of thumb rather than a set of user interface guidelines. This technique is based on the inspection in which evaluators are given some scenarios and are asked to explore the given interface using an identified set of 10 heuristics [19]. Practitioners had reported that heuristic evaluation allowed them to discover significant numbers of problems during various stages of design processes [20]. Moreover, it is also an inexpensive and a formative technique to evaluate interfaces. One of the advantages of using this usability inspection is the flexibility to adapt a predefined set of heuristics for a specialised domain [21,22]. The purpose of having these adapted heuristics is to discover usability problems in a system from the domain-specific perspective and fix them before end users start interacting with it. Researchers have adapted and created a specialised set of heuristics for various domains, and a brief description is as the following. In their research, Mankoff et al. [23] carried out informal surveys and pilot studies with the experts and created a modified set of heuristics for ambient displays. Baker et al. [24] created a new set of heuristics to evaluate collaborative technologies based on an existing framework called mechanics of collaboration [25]. This framework covers small-scale actions and interactions that group members must perform in order to get a task done in a collaborative fashion. Clarkson and Arkin [26] created a list of heuristics for human-robot interaction; they came up with an initial list of heuristics via brainstorming and synthesising the existing lists of potential heuristics. They also conducted pilot studies, consulted other domain experts, and used other informal techniques to finalise the initial heuristics. Kientz et al. [27] developed a set of derived heuristics for persuasive health technologies by reviewing the existing usability guidelines and related heuristics. Pinelle et al. [28] also used a similar approach to create heuristics for video games.

## Method

**Fig 1:**
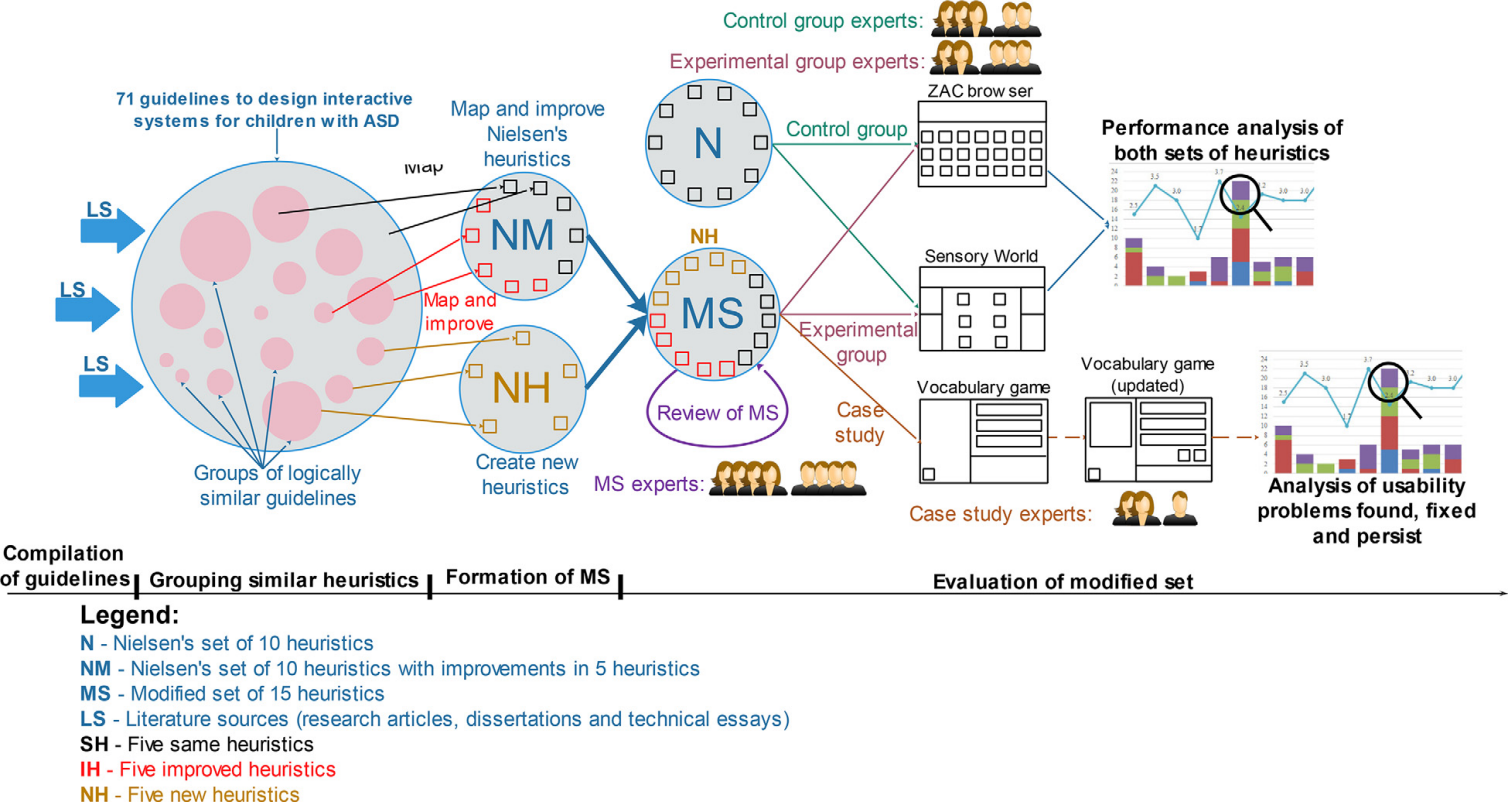
Processes carried out to develop heuristics for the evaluation of interactive systems for children with ASD.

In this research, we selected the existing guidelines in designing the interactive systems for children with ASD and used the heuristics of Nielsen to create a specialised set of heuristics for the evaluation of systems for these children. The user interface of an interactive system for children with ASD is important in the overall success of the system; the review of guidelines can reveal various guidelines which play a vital role in the design of user interface for these children. The key processes in this research consist of compilation of guidelines, grouping of similar guidelines, formation of a modified set of heuristics and its evaluation as shown in Fig 1. Each of these processes is described as follows.

### 3.1. Compilation of guidelines

A search on guidelines related to the design and development of interactive systems for children with ASD was carried out in different bibliographic databases; however, the search was restricted to research articles, dissertations, books and technical essays. Researchers of various studies had recommended guidelines for the design and development of systems for children with ASD, based on their research work on ASD. We compiled a list of the guidelines recommended by the researchers of the reviewed studies, three of which were found to meet our criterion. These relevant studies had used different terminologies (characteristics, features, or guidelines) in the design of systems for children with ASD. However, we will use the term ‘guidelines’ throughout this paper and will only consider those that are related to the design and development of an interactive systems for these children.

Higgins and Boone [29] compiled 47 guidelines (44 were related to design, whereas 3 were related to other aspects of the system) for the design of computer-assisted instruction (CAI) for children with ASD, using multimedia authoring application. Winograd [30] discussed 8 guidelines for designing a learning environment for children with ASD. Lastly, 18 guidelines were discussed in [31] on designing an application for children with ASD. Altogether, 70 guidelines (after exclusion of 3 guidelines) were finalised and are shown in 3 tables (S1–S3 Tables) with each table corresponding to one of the three above-mentioned studies.

### 3.2. Grouping similar guidelines

During the compilation process, some guidelines were found logically related to the design aspect described in the guidelines; therefore, the next step was to group these related guidelines using a process similar to that of an affinity diagram. A total of 29 groups were formed of which the first 22 groups (G1 through G22) comprised a total of 63 guidelines with each group having two or more related guidelines. Each of the remaining seven groups (G23 through G29) contained one guideline; all the groups and their associated guidelines are shown in Table 1.

**Table 1:** Group of similar heuristics.

Group	Guidelines
G1	1, 2
G2	3, 4, 5
G3	6, 7
G4	8, 9, 10, 11, 12
G5	13, 14, 15
G6	16, 17, 46, 50
G7	18, 19, 20, 21
G8	22, 23, 24
G9	25, 67, 68
G10	26, 27
G11	28, 29, 30
G12	31, 32
G13	33, 34
G14	35, 36, 37
G15	38, 39, 40
G16	41, 42, 43
G17	47, 48, 49, 52, 63
G18	53, 64
G19	57, 58
G20	59, 60
G21	55, 61, 62
G22	69, 70
G23	44
G24	45
G25	51
G26	54
G27	56
G28	65
G29	66

### 3.3. Mapping of guidelines and developing heuristics

This process involves mapping of guidelines to Nielsen’s heuristics and developing heuristics based on the outcomes of mapping. All the guidelines from the 29 groups were individually mapped to the Nielsen’s heuristics and the process of mapping started from G1 through to G29. This exercise revealed the extent to which the groups and their guidelines were mapped to the same heuristics of the original set. It also highlighted the groups and guidelines which were not mapped and required further necessary action to create new heuristics.

**Fig 2:**
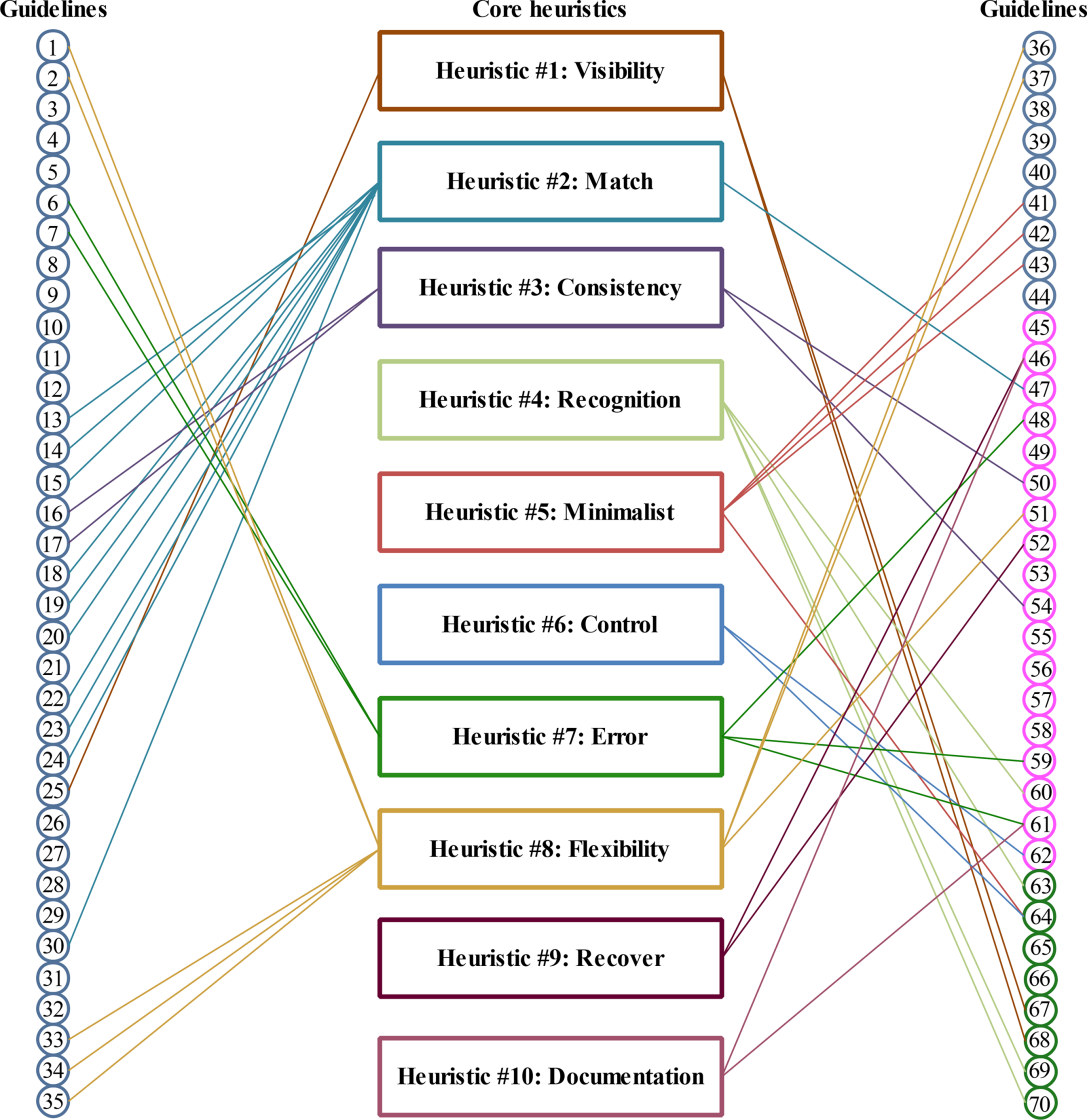
Mapping between guidelines and original heuristics.

Fig 2 shows 70 circles which correspond to the guidelines in Table 1; the groups are not shown in the figure in order to reduce complexity, i.e. to ensure the figure is not cluttered up with too much information. These circles are split into two halves with the first 35 circles placed on the left side, whereas the remaining 35 circles are placed on the right side. The colour of the circle indicates the source of the guidelines with circles in blue representing the guidelines by [29]; the guidelines given by [31] are marked in pink; and the guidelines from [30] are shown in green circles. All ten heuristics of Nielsen are placed in the centre, which are represented by rectangles and marked in a different colour. The lines between the circles and rectangles indicate that the guidelines and the heuristics are related to each other. The heuristics of Nielsen are improved when the corresponding guidelines match the context, but the heuristics do not cover some aspects of the guidelines mapped to them. The additional text in the heuristics is appended at the end of the heuristics and is written in *italics* to differentiate the new text from the existing text of the heuristics. The word ‘(improved)’ is also added to the names of these heuristics to distinguish them from the remaining heuristics of Nielsen.

The mapping process shows that (N = 43, 61%) of the 70 guidelines were mapped, whereas the remaining (N = 27, 39%) guidelines were not mapped to any heuristics of Nielsen. These unmapped guidelines are indicated by circles that do not have any line connected to them, for example Guidelines 3, 4, 5 and 8.

**Fig 3:**
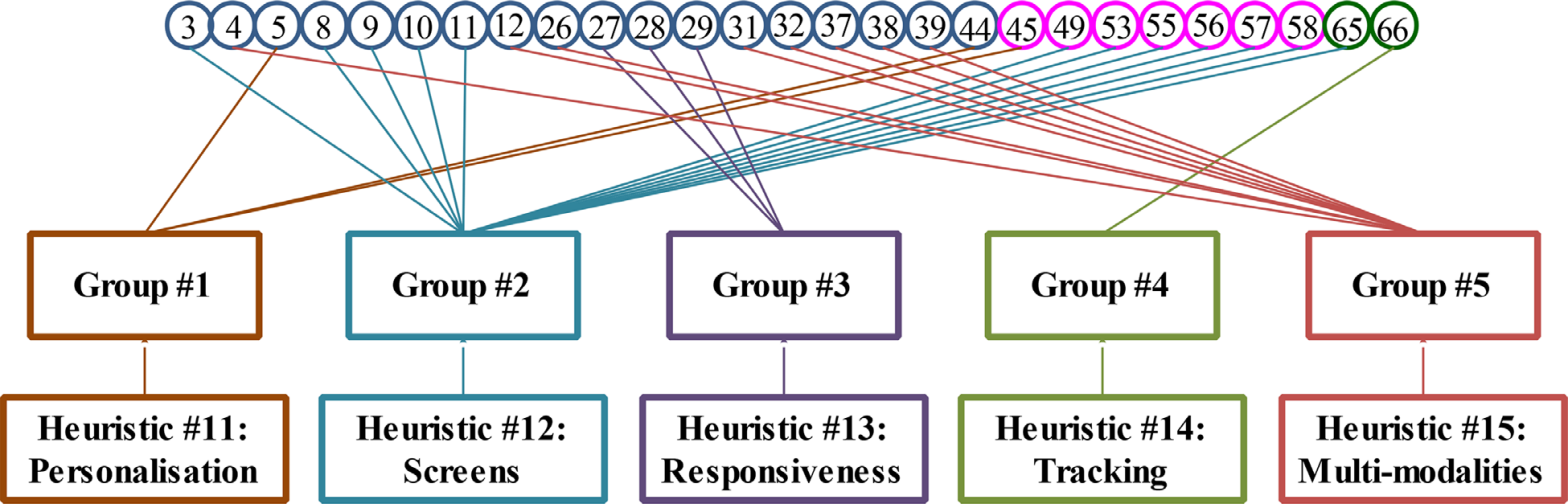
Grouping of remaining guidelines and creation of heuristics.

The remaining 27 guidelines are used to create new heuristics; these guidelines also represent certain aspects of the interface design which are not covered by the heuristics of Nielsen. We went through the remaining guidelines and regrouped related guidelines into 5 groups (i.e. Group#1 to Group#5) as shown in Fig 3. These five new heuristics were given names that indicated the gist of all the guidelines mapped to them: 1) personalisation of screen and action items; 2) user interface screens of the system; 3) responsiveness of the system; 4) state tracking of user actions; and 5) use of multi-modalities for communication.

Table 2 shows the modified set of fifteen heuristics. The first 10 heuristics of the modified set are from Nielsen’s heuristics; the first 5 heuristics are exactly the same whereas the other 5 heuristics are the improved heuristics. The last 5 heuristics are new and they are added to the modified set of heuristics. A single word written in the brackets ‘[]’ of each heuristic represents the shorter name of that heuristic.

**Table 2:** Modified set of heuristics.

No.	Heuristics
1	**[Visibility] of system status**
	The system should always keep users informed about the activities, for instance, what is going to happen, how long it will take, what is going on, through appropriate feedback within reasonable time.
2	**[Match] between system and the real world**
	The system should speak the users' language, with words, phrases and concepts familiar to the user, rather than system-oriented terms. Follow real-world conventions, making information appear in a natural and logical order.
3	**[Consistency] and standards**
	The system should use clear and consistent language so that users do not have to wonder whether different words, situations, or actions mean the same thing. Follow platform conventions in the design for consistency.
4	**[Recognition] rather than recall**
	Minimise the user's memory load by making objects, actions, and options visible. The user should not have to remember information from one part of the screen to another. Instructions for use of the system should be visible or easily retrievable whenever appropriate.
5	**Aesthetic and [minimalist] design—minimise distraction and keep design simple**
	The design of user interface screens should not contain information which is irrelevant or rarely needed as it may distract these children’s attention. Every extra unit of information in a dialogue competes with the relevant units of information and diminishes their relative visibility.
6	**User [control] and freedom (improved)**
	Users often choose system functions by mistake and will need a clearly marked ‘emergency exit’ to leave the unwanted state without having to go through an extended dialogue. Support undo and redo. *The system should allow users to move from one part to another and provide the facility to repetitively perform activities*.
7	**[Error] prevention (improved)**
	Even better than good error messages is a careful design which prevents a problem from occurring in the first place. Either eliminate error-prone conditions or check for them and present users with a confirmation option before they commit to the action. *If the users select wrong options, the system should provide alternative options for them to choose from*.
8	**[Flexibility] and efficiency of use (improved)**
	Accelerators—unseen by the novice user—may often speed up interactions for the expert user such that the system can cater to both inexperienced and experienced users. Allow users to tailor frequent actions. *The system should carry out initial evaluation of performance to identify the level of the user and suggest an appropriate activity to start with*.
9	**Help users recognise, diagnose, and [recover] from errors (improved)**
	Error messages should be expressed in plain language (no error codes), precisely indicate the problem, and constructively suggest how to avoid this error. *The system should provide multimedia demonstration to provide suggestions to the user when an error occurs*.
10	**Help and [documentation] (improved)**
	Even though it is better if the system can be used without documentation, it may be necessary to provide help and documentation. Any such information should be easily available to the user, focus on the user's task, *provide multimedia demonstration* of tasks to be carried out, and not be too large.
11	**[Personalisation] of screen items**
	The system should allow personalisation of screen items based on needs, abilities and preferences of an individual child. Screen items should be large enough for children to read and interact with. It should also allow them to change various settings of system background, font, colour, screen size and others.
12	**User interface [screens] of the system**
	The change on the screens of user interface of the system should take place step-by-step as children with ASD will not be able to cope with sudden or drastic changes made.
13	**[Responsiveness] of the system**
	Each action performed by children with autism (for instance click and select) should have no latency as children with autism have shorter attention span, typically forget quickly and can easily get frustrated.
14	**[Track] user activities monitor performance and repeat activity**
	The system should keep a history of all the activities performed by the user, time spent, responses provided, results and others. They should be allowed to view their performance over a period of time and can return to any of the past activities to repeat it.
15	**Use of [multi-modalities] for communication**
	Users should be given the option to use different devices to provide input to the system. The communication between users and the system should take place using multimedia (text, digitised audio, images, animation, video and others).

### 3.4. Evaluation of modified set

The modified set was evaluated through three different studies. An expert review study was conducted to evaluate the heuristics in the set and provide suggestions so that heuristics could be improved. After incorporating the necessary changes in the heuristics based on the recommendations of the experts, we also wanted to ascertain the effectiveness of the modified set of heuristics in the identification of usability problems among interactive systems for children with ASD. The effectiveness of the set was measured by performing an evaluation on an existing interactive system (usability study 1) and a new system being developed (usability study 2). The details of all three studies are described further below.

#### 3.4.1. Expert review study

Expert reviews are widely used as a way to evaluate and improve the quality of the framework and software developed or to test their functionalities [32]. They have also been used to evaluate and improve the heuristics developed for the purpose of assessing a specific product. The approach of using expert reviews is also adopted in this research to evaluate the modified set of heuristics and the experts in the area of HCI were recruited. The inter-rater reliability (IRR) analysis was performed to gauge the consistency in the categorical ratings of the questions among the experts. The details of expert review study are discussed in Section 4.

#### 3.4.2. Usability study 1

The effectiveness of this modified set of heuristics in identifying usability problems was measured by comparing the results of usability problem identification between the modified set of heuristics with Nielsen’s set of heuristics. We wanted to determine the following:

1. Total number of usability problems identified and a comparison of the average severity ratings of both groups (control and experimental) between the two systems.
2. Contribution of the 5 new heuristics in terms of problem identification and a comparison of the average severity ratings between the two systems.
3. Impact of the 5 improved heuristics on the two systems in comparison with the corresponding heuristics in Nielsen’s set.
4. The frequently violated heuristics in the original and modified sets of heuristics.

Hypothesis tests were also conducted for the statistical validation of results to achieve objectives a, b and c of this usability study. Two existing interactive systems were selected; experts were recruited and divided into two groups, i.e. the control group which was given Nielsen’s set of heuristics and the experimental group which was given the modified set of heuristics. The consistency among the experts and their responses in each heuristic were measured through the intra-class correlation (ICC). ICC is a commonly used statistical test which can be applied to assess IRR for ordinal, interval, and ratio variables. The details of the tests in this study are presented in Section 5.

#### 3.4.3. Usability study 2

The effectiveness of the modified set of heuristics to identify usability problems in a system being developed was investigated in two phases; in the first phase, usability problems were identified while in the second phase, an analysis was conducted to measure the extent to which the identified problems had been fixed before the final version of the system was ready for children with ASD to use. The details of this usability study are discussed in Section 6 below.

## Expert Review of the Modified Set of Heuristics

This section describes the participants and the recruitment process, instrument used in the study, study protocol, inter-rater reliability (IRR) test and the results of this expert review study.

### 4.1. Participants and recruitment

The experts selected for this study include academic staff who teach HCI and also conduct research in the same area; they have worked with children and have the relevant experience in heuristic evaluation. The search for these experts was performed in Microsoft academic search portal (http://academic.research.microsoft.com/) and Google search engine(http://www.google.com). Based on the search results, thirteen experts were identified for this study. The email invitations were sent to these experts and seven of them replied in the affirmative, confirming their participation in this study. The recommendations from these experts were also taken into consideration, and this involved inviting more experts. One expert was contacted based on these recommendations and had accepted our invitation. Four of these experts are currently conducting research in the area of children with ASD. The profiles of these experts are shown in Table 3.

**Table 3:** Demographic profiles of experts.

Expert#	Gender	Experience	University/Institution
1	Male	6 years	University of Maryland Baltimore County (UMBC)
2	Female	11 years	University of Malaya (UM)
3	Female	3+ years	University of Maryland Baltimore County (UMBC)
4	Male	10 years	University of Central Lancashire (UCLan)
5	Male	10+ years	Vienna University of Technology
6	Male	10+ years	University of Iowa
7	Female	5 years	UCL Institute of Education
8	Female	15 years	Multimedia University (MMU)

### 4.2. Instrument used

The main instrument used in this study is a questionnaire containing 7 questions as shown in Table 4. From Q1 to Q4, the experts were asked to evaluate the relevance, clarity and relation of each heuristic and if the heuristic required any additional information. The experts were asked to provide their opinions using one of the three options (agree, disagree or not sure). The information about the missing heuristic was obtained through Q5 to Q6. The aim of Q5 is to verify if there is any missing heuristic with three options provided (yes, no, not sure), and the purpose of Q6 is to provide details of heuristic that are missing in the set. Q7 allows the experts to give remarks, further explanation and clarification for each heuristic. This feedback is important for us to better understand the experts’ views. The descriptions of these questions are presented in Table 5.

**Table 4:** Questions and options.

Q#	Text	Options		
1	Is heuristic relevant?	Agree	Disagree	Not sure
2	Is the description of heuristic clear?	Agree	Disagree	Not sure
3	Does the name and description of heuristic match each other?	Agree	Disagree	Not sure
4	Does it require more details to be added?	Agree	Disagree	Not sure
5	Is there any heuristic that is missing?	Yes	No	Not sure
6	Missing heuristic (if any):			
	Name of the heuristic: ____________________________________________________			
	Description of the heuristic: _______________________________________________			
	______________________________________________________________________			
7	Remarks (if any): ________________________________________________________			
	______________________________________________________________________			

**Table 5:** Descriptions of questions.

Q#	Focus of question	Description
1	Relevance	To determine if a heuristic can help to identify issues in the design of interactive systems for children with ASD.
2	Clarity	To understand if the description of a heuristic is easy to read and interpret; and whether all the details mentioned in the description are related to the heuristic.
3	Relation	To confirm if the name and description of a heuristic are related to each other.
4	Additional details	If there are any additional details missing in the name or description of a heuristic.
5	Missing heuristic	If there is any heuristic missing in the modified set that can be helpful in finding usability problems in the systems for children with ASD.

### 4.3. Study protocol

As part of this study, each reviewer was independently contacted through Skype videoconferencing to perform face-to-face evaluation of the heuristics. During the session, the expert was briefly informed about the background of study and its objectives and was introduced to the modified set of heuristics. Informal discussion took place before the experts were asked to answer all the questions in the instrument and submit the text through email. The consistency of the opinions of the experts was analysed using IRR.

### 4.4. Data analysis

#### 4.4.1. Measure the frequency of the opinions provided by the experts

The data provided by the experts were analysed by using the frequency of responses in relation to the 4 questions (Q1 to Q4) of Table 4. The frequency was calculated as a sum of responses for ‘agree’, ‘disagree’ and ‘not sure’ among the 4 questions.

#### 4.4.2. Measure the consistency across experts using IRR

The first 3 questions of Table 4 pertain to the rating of heuristics from the experts’ viewpoint; the data obtained from these questions were stored as nominal variables. There are various common methods that can be used to carry out IRR analysis of nomial data; these include Cohen’s Kappa [33], Light’s Kappa [34] and Davies and Fleiss’s Kappa (DF Kappa) by [35]. Cohen’s Kappa analyse the IRR of only two experts’ data [36] and therefore, in this study, the strategy to integrate all the experts’ consistency was adopted from Light and DF Kappa methods to compute Kappa for the 8 experts.

#### 4.4.3. Analysis of comments provided by the experts for each heuristic

All the comments provided by the experts were classified as suggestions that made information more readable and easy to understand, disagreements related to heuristics, or concerns by any experts that required attention. We addressed all the comments individually, and actions (add, edit or remove information) were taken based on the suggestions provided by the experts.

### 4.5. Results

#### 4.5.1. Frequency of the opinions provided by the experts

The results of frequency analysis are not shown in this subsection so as to reduce duplication of IRR data presented in Section 4.5.2. The analysis shows the details below:

1. For most of the heuristics, the experts agreed that they were relevant, the descriptions were clear and there exist a relationship between the names and descriptions.
2. There were four heuristics (1, 8, 9 and 15) in which 4 or more experts had shown disagreement that the descriptions of heuristics were sufficient; similarly, 4 or more experts indicated that the name and description of heuristic 14 did not match. The comments related to these five heuristics were analysed and the discussion is presented in Section 4.5.3.

#### 4.5.2. Consistency across experts using IRR

The results of Cohen’s Kappa for all pairs of experts show consistency between the experts’ pairs at the level of 0.05 (see S4 Table). We calculated the average mean values to obtain the Light’s Kappa as the final consistency index involving all the experts. The resulting kappa indicates a fair consistency (p-value < .05, k = .4), and this is in line with the estimates of IRR obtained from the coding of similar constructs in previous studies. The strong consistency (on answer = 1, i.e. ‘Agree’) is found among the experts for heuristics 10 and 11. The consistency is reduced by a modest amount of error variance due to difference in the rating of different experts: i) heuristics 5, 6, 14 and 15 for question 1, ii) heuristic 14 for question 2 and iii) heuristic 15 for question 3. However, the IRR analysis shows that experts have a consistency in their ratings on all the questions. These ratings are considered as adequate to conduct studies 1 and 2 in this research which are described in Sections 5 and 6.

#### 4.5.3. Comments provided by the experts for each heuristic

Table 6 shows 13 comments for the five heuristics (1, 8, 9, 14 and 15) mentioned in Section 4.5.1; the majority of the comments are classified as suggestions from the experts. The experts have provided suggestions only for 3 heuristics (1, 14 and 15), but they have provided a mixture of comments (suggestions, concerns and disagreements) for the remaining 2 heuristics (8 and 9).

**Table 6:** Comments of experts and the actions performed.

Heuristic	Comments by the experts	Classification	Actions performed
1	The word Visibility is quite ambiguous. Perhaps can explain ‘visible’ in terms of so and so. System status is also not so clear. Can you provide an example for system status?	Suggestion	We have updated and modified the description of this heuristic.
1	Particularly predictability is very important for many children with ASD. So, provide a plan beyond the current status, e.g., what will happen, when will it finish etc.	Suggestion	This information has been incorporated in the heuristic.
1	You might want to think about the form of feedback which could be appropriate for children with ASD such as visual	Suggestion	This is mentioned in heuristic 15 that the system should use multiple modalities for communication with the user.
8	I’d say the important thing is personalisation, no matter how it’s accomplished (it could be through the system, it could be based on the child’s choices, or on what an adult who knows the child thinks will work best). Better to say what your goal is (personalisation), than to say specific ways of getting there.	Suggestion	This heuristic focuses on finding the correct level of user with respect to contents available in the system by conducting a small-scale relevant test. It is different from heuristic 11 in which a user is allowed to change settings.
8	Very difficult to do by a system. Every assessment of autism is by definition crude and may not help the system to adapt its behaviour. Autism is too unpredictable for this adaptation to work properly, also because too little can be sensed by machines (emotional state, for example)	Concern	There are various systems which provide multiple levels of activities (for instance, easy, medium, hard among others) for users to choose from. The purpose of this heuristic is to check if the system is flexible enough to test the level of users and provide recommendation according to the level.
8	Suddenly moves from being general to being about educational software. This heuristic was not originally referring the level of educational content but the interface design.	Suggestion	The context of this heuristic has been changed from the educational level to general.
9	Aim for error-free systems	Suggestion	No changes are made in the heuristic but developers should try to ensure that chances for errors to occur are minimal as children may feel frustrated easily.
9	I think this means Help Functions similar to what contains in Heuristics no 10. Perhaps can combine these as one heuristic	Suggestion	This heuristic is about how children can be informed of what has happened and how they can avoid these errors in the future. On the contrary, heuristic 10 is about providing multimedia based help and documentation than just the text.
9	Good language is important, but more importantly, is it to reassure children with autism, make them feel that they know what will happen. I am unconvinced, that multi-media makes a whole lot of difference	Disagreement	It may be difficult for these children to interpret text-based errors. These children are visual learners and showing them visually how errors can be avoided will be more effective than the former method.
9	How would it suggest a suitable solution?	Concern	Based on the type of error that has occurred, the system can inform children why this error has occurred and how they can avoid this error in the future. The description of the heuristic has been changed to reflect a means of avoiding errors rather than providing solution.
14	It needs clarification. You want to provide feedback on the long-term use of a system, both for children and adults.	Suggestion	The description of this heuristic has been modified to convey better understanding. The purpose of this heuristic is to see if the system retains information across multiple sessions so that long-term performance analysis can be provided to these children.
15	Clarify if this is for one-way or two-way communication. Also, look up the term multi-modal, which works better in this case (I think)	Suggestion	This heuristic is more towards providing multiple modalities for a child to interact with the system. Therefore, the term multimedia is changed to multi-modalities in the heuristic.
15	Depending on the child, too much choice might be difficult for them, and too much multimedia is too distracting.	Suggestion	This concern needs to be taken into consideration when designing an interactive system for these children.

## Usability Study 1 –Evaluation of Existing Interactive Systems

This section describes the participants recruited, instruments used, protocol used, analysis of data, followed by presentation and discussion of results.

### 5.1. Participants

We recruited ten participants for this study, including researchers and academic staff working in the field of HCI or interface design and its evaluation. The demographic information of these participants is given in Table 7.

**Table 7:** Demographic information of participants.

Participant#	Gender	Age	Years of experience	Expertise
1	M	33	1	Researcher
2	F	33	4	Academic staff
3	F	32	5	Academic staff
4	M	30	1	Researcher
5	F	32	3	Researcher
6	M	31	2	Researcher
7	M	42	3	Academic staff
8	F	35	6	Academic staff
9	F	30	1	Researcher
10	M	32	2	Researcher

### 5.2. Instruments used

Two interactive systems for children with ASD were evaluated by the experts; the first system chosen was a ZAC browser named ‘Zac Browser’ (http://zacbrowser.com/), specifically developed for children with ASD. The second system was a Sensory World website ‘Sensory World’ (http://www.sensoryworld.org/), designed for children to practise and learn various basic skills and activities on their own at home. These two systems were chosen based on the following criteria:

1. Systems can be used online or available for free download
2. Children with ASD can use the systems with minimal guidance
3. The systems can operate on Microsoft Windows platform

Both of the above criteria, the chosen systems are representative examples of interactive systems for children with ASD and are not crucial for the study to be carried out. The experiments of this study can also be replicated in other systems and both sets of heuristics can be applied for the purpose of evaluation.

#### 5.2.1. ZAC Browser

ZAC is the very first web browser specifically developed for children with ASD. It allows children to play games, watch video clips in Television section, listen to music, read different stories, carry out various activities and use available applications within the boundaries of the web browser while acquiring and enriching their knowledge in an enjoyable manner. This browser is referred to as System 1 in the remaining parts of this paper.

#### 5.2.2. Sensory World

Sensory World is a website designed based on a house and garden setting. Children have the opportunities to explore different rooms with hands-on experience while interacting with the items in a specific room. This website allows each child to learn various basic skills while having fun with a range of stimulating and appropriate activities. For instance, the activities include decorating your own room by selecting and placing items at suitable places; learning the importance of health & safety; learning about food hygiene in the kitchen and other places; and taking care of nutrition for the body. This website is termed System 2 in the remaining sections of the paper.

### 5.3. Study protocol

The following steps were carried out as a part of the study protocol:

1. Participants were invited through email; they were informed that evaluation data submitted would remain anonymous, and they were requested to respond to the email concerning their willingness to be part of the evaluation process. Participants who accepted the invitation were randomly assigned to a control or an experimental group.
2. Each group (control and experimental) consisted of five participants. Participants in the control group were given the original set of heuristics whereas the modified set of heuristics was given to the experimental group for the evaluation of the two systems.
3. Participants carried out evaluation at their own site. A briefing session was held via Skype to inform participants about a few aspects of the study: i) purpose of evaluation; ii) systems to be evaluated; iii) set of heuristics (original or modified) to be used; iv) exploration of the systems; v) identification of usability problems; and vi) submission of data through email. During the evaluation, for each heuristic, participants were asked to write a brief description of all the problems related to that category together with severity ratings between 0 and 4, where 0 corresponded to ‘not a problem’; 1, ‘cosmetic problem’ and 4, ‘usability catastrophe’.

### 5.4. Data analysis

#### 5.4.1. Measure the consistency in the problem identification of the experts

Before conducting this evaluation, we first analysed the IRR of the experts to test the consistency in the problem identification. Since the number of problems identified by the 10 experts in all the heuristics involved ratio variables, Intraclass Correlation Coefficient (ICC) [37] was used to measure the consistency of the experts. Unlike Kappa, which quantifies IRR based on agreement or no agreement among the experts, the ICC considers the difference between the values of consistencies among the experts to compute the IRR estimate. This indicates that a lower difference among the experts, i.e. low inconsistency will result in a higher value of ICC while a larger difference, i.e. high inconsistency will result in a lower value of ICC.

#### 5.4.2. Measure the effectiveness of the modified set of heuristics

The details of data analysis are described below.

Measure the overall results of the modified set of heuristics: The analysis of overall results is based on the following two parameters:

1. Number of usability problems found: it is calculated as a sum of all the problems identified by the experts in each heuristic of the original and modified set for System 1 and System 2.
2. Average severity ratings: the average severity rating of the experts is calculated for all the problems identified in each heuristic of the original and modified set for System 1 and System 2.

Measure the contribution of the 5 new heuristics: The analysis to measure the contribution of the 5 new heuristics is based on 3 parameters; the first 2 parameters are the same as above (i.e. ‘number of usability problems found’ and ‘average severity ratings’) while the third parameter is ‘percentage of problems identified’. The value of this third parameter indicates the percentage of problems identified by the 5 new heuristics in comparison with the total number of problems identified by the modified set of heuristics.

Measure the impact of the 5 improved heuristics: The analysis to measure the impact of the 5 improved heuristics is based on both parameters, i.e. ‘number of usability problems found’ and ‘average severity ratings’.

Identify the frequently violated heuristics in the original and modified sets of heuristics: The analysis to identify frequently violated heuristics between the original and modified sets was performed using a parameter ‘number of usability problems found’ for System 1 and System 2.

#### 5.4.3. Hypothesis to analyse effectiveness of the modified set

The following three hypothesis tests were conducted to statistically validate the outcomes from Section 5.4.2. The list of variables used for the hypothesis tests includes:

1. Number of problems identified (dependent, ratio)
2. Experts (independent, nominal)
3. System (independent, nominal)
4. Sets of heuristics (independent, nominal)
5. Severity (independent, nominal)

H1 –The modified set of heuristics is more effective than the original set in problem identification: The heurisitics of the modified set were compared with the heusitics in the original set using a form of repeated measure. The Analysis of Variance (ANOVA) was used to test the differences between the modified and original set of heuristics. The test of differences between groups of heuristics was conducted repeatedly while the other conditions such as the systems and the experts remained the same and hence, we applied the Split-plot ANOVA (SPANOVA). The SPANOVA is used to test the differences between two or more independent groups whilst subjecting participants are repeatedly measured [38]. There are certain assumptions that needs to be taken into account when performing SPANOVA test:

1. Each sample is independently and randomly selected.
2. The distribution of the response variables follows a normal pattern. The Kolmogorov-Smirnov test [39] is conducted to verify that the sample comes from a known population and has normal distribution.
3. The population variances are equal for all responses at the group levels. The significance of results is checked through P-value at the level of 0.05.

SPANOVA involves modelling of data using a linear model [40] which is implemented in statistical packages such as SPSS.

The heuristics in both the original and modified sets have been grouped as following for the purpose of statistical analysis:

1. First 5 heuristics in the original set
2. Second 5 heuristics in the original set
3. First 5 heuristics in the modified set
4. Second 5 heuristics in the modified set (improved heuristics)

We used SPANOVA to examine the rate of problem identification by 4 independent groups under the same conditions (systems and experts). For this hypothesis, Groups 1 and 2 were merged and termed the ‘original set’ while Groups 3 and 4 were merged and termed the ‘modified set’.

H2 –The 5 improved heuristics in the modified set will be able to identify a higher number of usability problems than the corresponding heuristics in the original set: The analysis of the 5 improved heuristics was based on the same variables from Section 5.4.3 and groups formed in H1; the SPANOVA test was used to examine the rate of problem identification between Groups 2 and 4.

**Fig 4:**
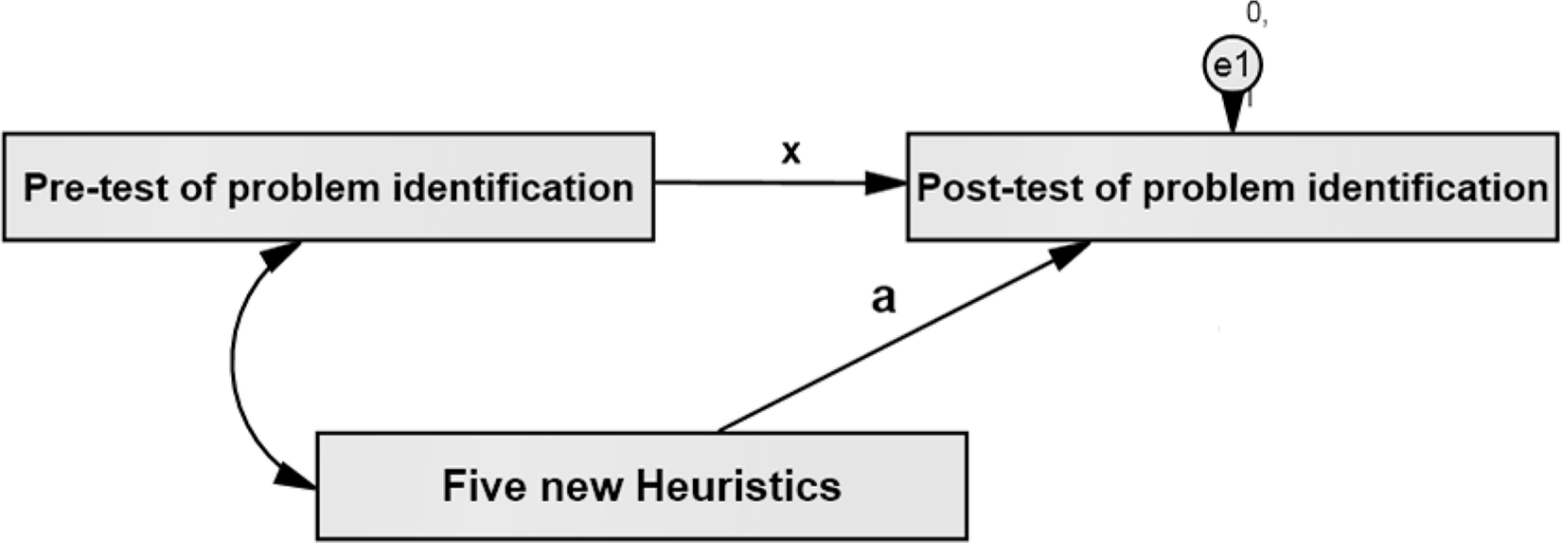
Hypothesis model.

H3 –The 5 new heuristics will contribute to the identification of problems using the modified set: A repeated measurment test was conducted to determine the effectiveness of the five new heuristics. In the pre-test, we measured the problems which were identified by the original set as well as the first ten heuristics of the modified set (five original and five improved heuristics). In the post-test, we measured the problems identified by all the fifteen heuristics of the modified set. The pre-post test anlysis would reveal the effectiveness and contribution of the five new heuristics in the overall problem identification. We used structural equation modelling (SEM) to create a model of data and a mediated method [41–43] to prove the effectiveness of the new heuristics. For the mediated method, the model as shown in Fig 4 is analysed with the assumption a = 0 (null-hypothesis), where a is the degree of effect of the new huristics on the post-test. If the p-value < .05, CFI< .90 and RMSEA > .08 then the model is not fit to collect data and the assumption is incorrect [44,45]. In this condition, the null hypothesis is rejected and the effectiveness of the new heuristics is proven.

We also analysed the model with the value of Chi square (CMIN) when a≠0 [46]. If the value of Chi square in the model, by considering the effect of new heuristics is smaller, then it is proven that the new heuristics affect the problem identification.

### 5.5. Results

The overall results of the evaluation carried out are presented first, followed by the answers to the 3 hypotheses.

#### 5.5.1. Intra-class Correlation Coefficient Results (ICC)

The results of ICC are presented in S5 Table; the results show that the average measure ICC is .780 with a 95% confidence interval from .198 to .984 (F(3,27) = 4.550, p< .05). This indicates that the experts have a high degree of consistency. The high value of ICC shows that a minimal amount of measurement error was introduced by the independent experts, and therefore consistency for subsequent analyses is not substantially reduced. The ratings of usability problems are deemed suitable for further analysis in this study.

#### 5.5.2. Overall results of both sets/results of Nielsen’s heuristics

**Fig 5:**
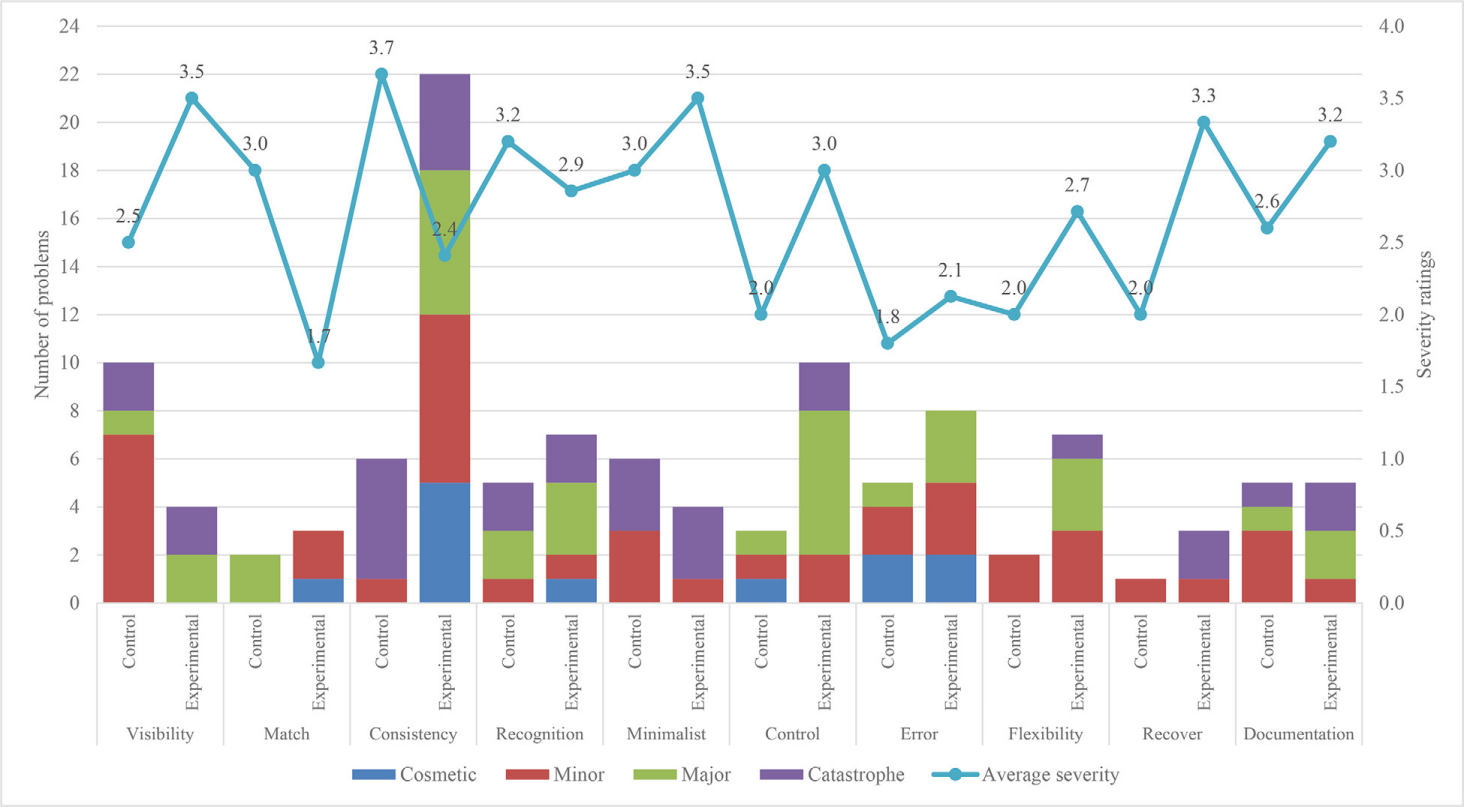
Number of usability problems and average severity ratings found in System 1 by both groups.

**Fig 6:**
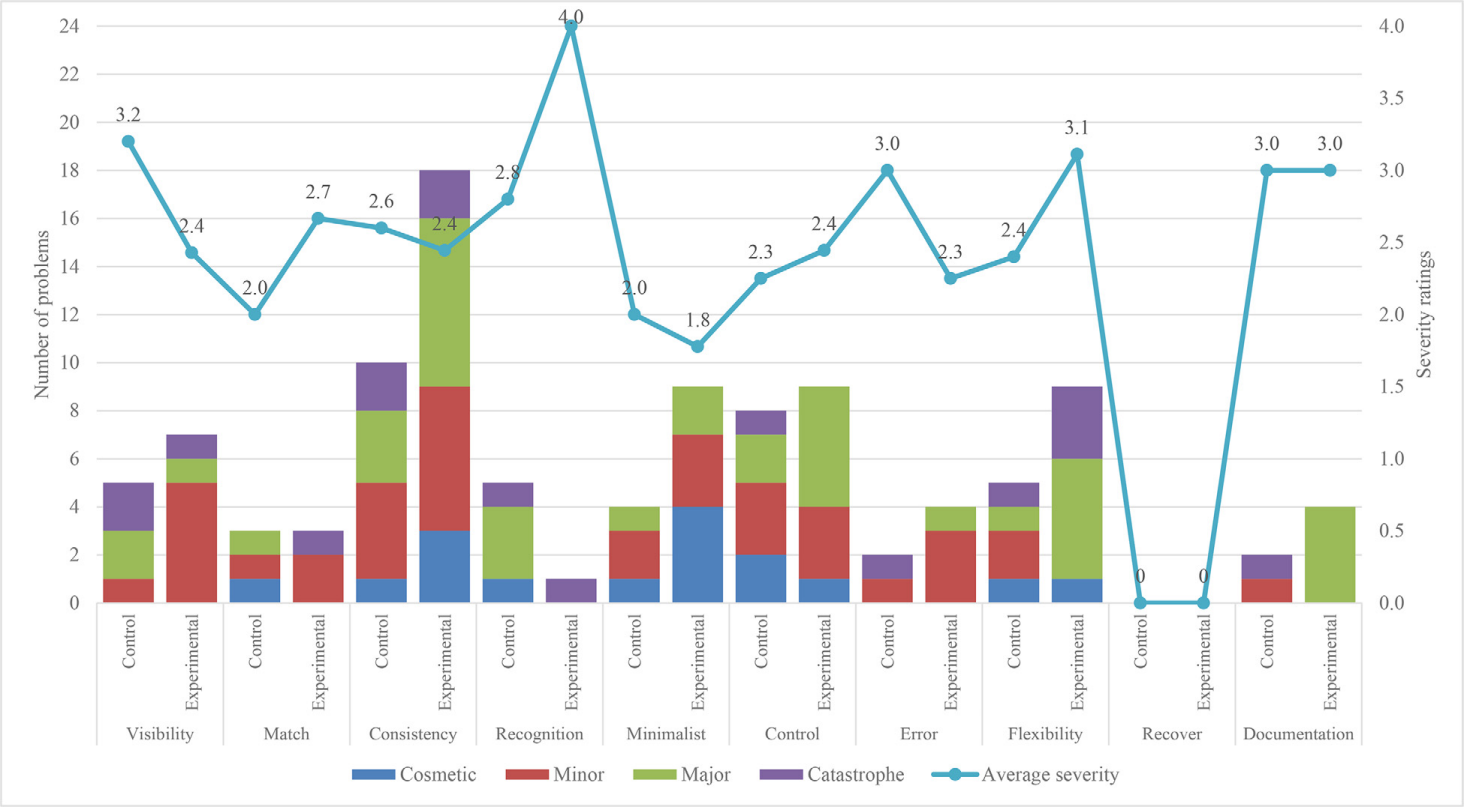
Number of usability problems and average severity ratings found in System 2 by both groups.

Figs 5 and 6 show the number of problems found, and the average severity of all the problems occurring in System 1 and System 2 using the original set and the corresponding first 10 heuristics of the modified set. The first 5 heuristics are the same as those in Nielsen’s original set, whereas the last 5 heuristics are Nielsen’s improved heuristics. The problems encountered by the control and experimental groups for each heuristic are placed together. For each heuristic, the stacked column shows the number of problems found in four severity ratings (cosmetic problem, minor usability problem, major usability problem and usability catastrophe), whereas the line connecting the markers shows the average severity ratings of all the problems found. A single-word name in each column represents the shorter name of heuristics from Table 8.

**Table 8:** Number of problems and average severity rating using different types of heuristics in both of the systems reported by control and experimental groups.

	System #1				System #2			
	Control		Experimental		Control		Experimental	
Heuristics	# of problems	Average severity	# of problems	Average severity	# of problems	Average severity	# of problems	Average severity
**Same**	29	3.1	40	2.8	27	2.5	42	3.0
**Improved**	16	2.1	33	2.9	17	2.1	29	2.1
**New**			19	2.9			14	2.5
**Total (10 heuristics)**	45	2.6	73	2.8	44	2.3	71	2.5
**Total (15 heuristics)**			92	2.8			85	2.5

The total number of problems and their average severity in System 1 and System 2 as reported by the control group and experimental group are as shown in Table 8. This table shows a slight improvement in the results of the experimental group. This variation is due to two reasons: 1) the interpretation of problems found by the evaluators; and 2) the addition of description in the 5 improved heuristics of the modified set. The analysis of the improved heuristics is presented in Section 5.5.4. The average severity ratings of all the problems in both of the systems by the control group show that they are minor, whereas the average severity ratings of the problems found by the experimental group show that most of the problems are major.

#### 5.5.3. Contribution of 5 new heuristics

**Fig 7:**
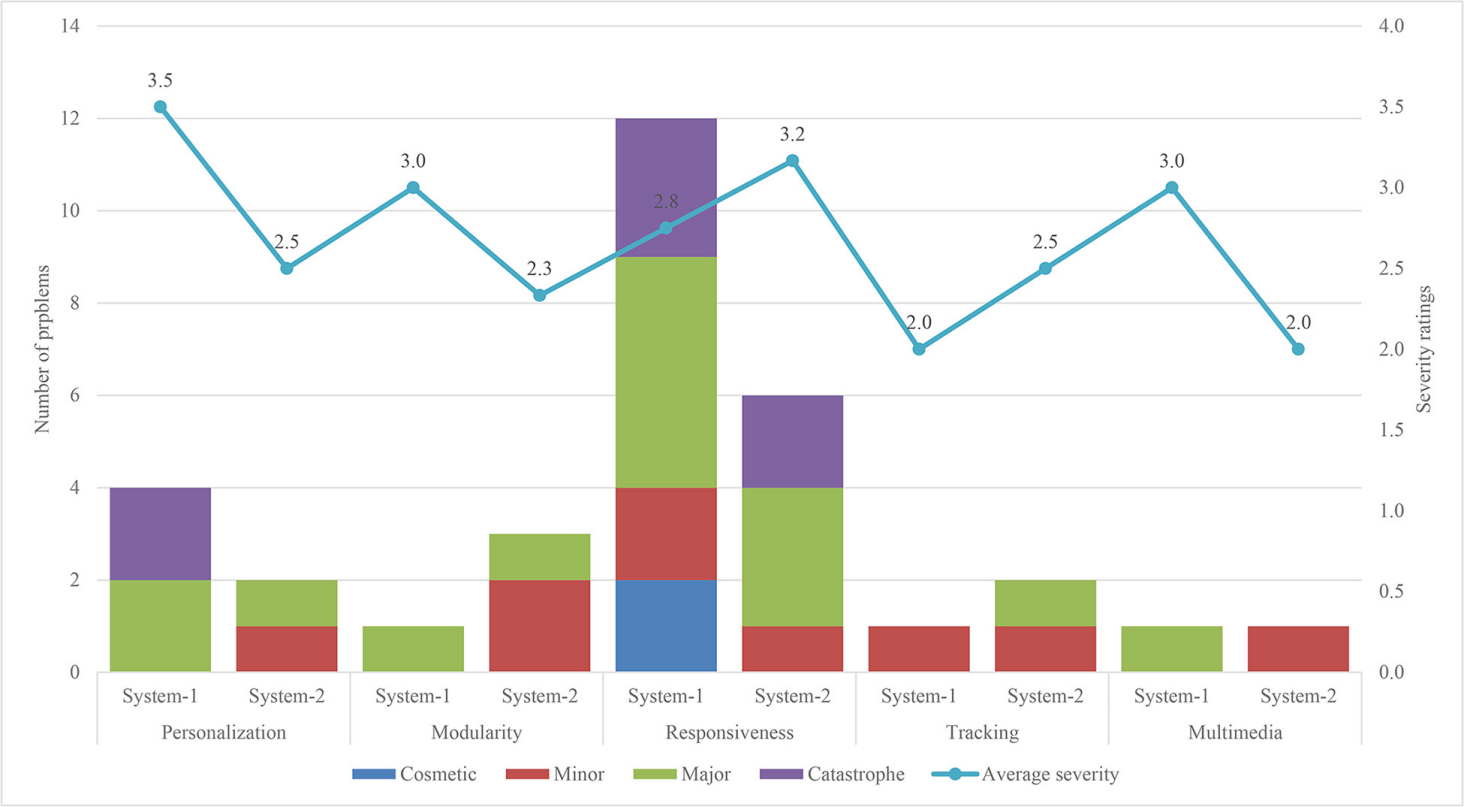
Number of usability problems and average severity ratings found in System 1 and System 2 using 5 new heuristics of the modified set.

Fig 7 shows the usability problems found and the average severity rating in System 1 and System 2 using the 5 new heuristics of the modified set. These pieces of information for the two systems are placed side by side under the same heuristic. These five new heuristics found (N = 19, 22%) of the total number of the usability problems in System 1 through the modified set. This shows that about a quarter of the usability problems in the experimental group were found by these heuristics. Among these five new heuristics, responsiveness heuristic was violated (N = 12) times compared with the others.

These five heuristics found (N = 14, 18%) of the total number of the usability problems in System 2 through the modified set. This shows that almost one-fifth of the usability problems in the experimental group were found by these heuristics. In the five new heuristics, the highest number of problems (N = 6) was found in responsiveness heuristic. The severity average ratings of both systems show that problems found by these 5 new heuristics are major.

#### 5.5.4. Impact of the 5 improved heuristics

The results for both systems show that the experimental group found slightly more problems than the control group. This shows that the added description in the improved heuristics had allowed evaluators to find an additional number of problems pertaining to children with ASD. Table 9 shows examples of problems identified by the evaluators in both of the systems using these 5 improved heuristics. Based on the average severity ratings in Fig 5 for System 1, the results are mixed. For both systems, except error heuristics, the severity of problems found by the experimental group is slightly higher than that of the control group. The results for System 2 in Fig 6 show that minor problems were found by both of the groups.

**Table 9:** Examples of problems found using 5 improved heuristics.

System	Heuristic	Sample problem found
System 1	Documentation	There is no documentation provided in the games on what needs to be done and how the game is to be played.
	Flexibility	It is difficult to differentiate between main icons and sub-icons on each page.
	Recover	The system does not show any error message but hangs indefinitely if a user unintentionally clicks on a wrong option.
System 2	Error	In ‘exploring the garden’, clicking on multiple explorers generates various sounds at the same time, which can be difficult for user to understand.
	Error	In the lounge area, once user has completed the decoration, nothing else happens.
	Documentation	A user needs to click on PLAY button continuously to increase the volume of music in the music room and no such instructions are given.

#### 5.5.5. Frequently violated heuristics in the original and modified sets of heuristics

The analysis of the frequently violated heuristics is based on Figs 5, 6 and 7, and the comments given by the evaluators. The frequently violated heuristics include responsiveness, consistency, minimalist and control. Responsiveness heuristic was more frequently violated than the remaining four heuristics of the modified heuristics. This finding indicates that the response of the systems is important to keep the attention of users; otherwise, they may be distracted by other interactive systems installed on the computer. There are various inconsistencies in the systems that may distract the users, and this is evidenced by the number of times the heuristic was violated. Another frequently violated heuristic is minimalist. The analysis of problems related to this heuristic shows that there are a few places in the systems where the users are shown a lot of unnecessary information. These children could be overwhelmed by the large amount of information, which might slow them down in using the systems. The analysis of the control heuristic shows users could not move freely from one part to another in the systems or repeat activities as many times as they like. Examples of problems identified by the evaluators in the above-mentioned four heuristics are shown in Table 10.

**Table 10:** Examples of problems found by frequently violated heuristics.

Heuristic	System	Sample problem found
Responsiveness	System 1	In the majority of the screens, the system takes a few seconds to respond upon clicking.
	System 2	A mouse icon on the list of components indicates a clickable item, whereas on other screens, a circle icon shows that it is a clickable item. Such inconsistencies may confuse users in using the system.
Consistency	System 1	The system does not have ‘minimise’, ‘restore’ and ‘close’ buttons, which are available in all typical systems.
	System 2	On the login screen, pressing enter key after inputting username and password does not verify information provided.
Minimalist	System 1	In the games section, there is a house on the right side of the main screen; the colour of the house is white and other colours are included as well. With the white colour of the house, it is difficult to read the names of icons in front of the house, namely ‘Buried Treasure’ and ‘Chill for Boowa’.
	System 2	The items in the bedroom and lounge are placed too close to one another, and the audio clip is continuously and repeatedly played as mouse is moved over those items.
Control	System 1	The system should let the user perform the tasks one after another to ensure they understand the activity properly.
	System 2	In the kitchen, if a user mistakenly changes some setting pertaining to specifying nutrition for the entire week, there is no way to undo it.

#### 5.5.6. Hypotheses results

H1 –The modified set of heuristics is more effective than the original set in problem identification: The analysis of SPANOVA results shows that there is an interaction effect between *Sets of heuristics* and *Severity* [F(3, 16) = 1.327, p< .05]. The results of tests for subject effects indicate that there are significant differences in the problem identification between the two systems using both sets of heuristics [F(3, 16) = 4.03, p< .05; mean difference = 2.55]. Therefore, the null hypothesis is rejected and the modified set of heuristics is significantly effective in facilitating the problem identification.

**Fig 8:**
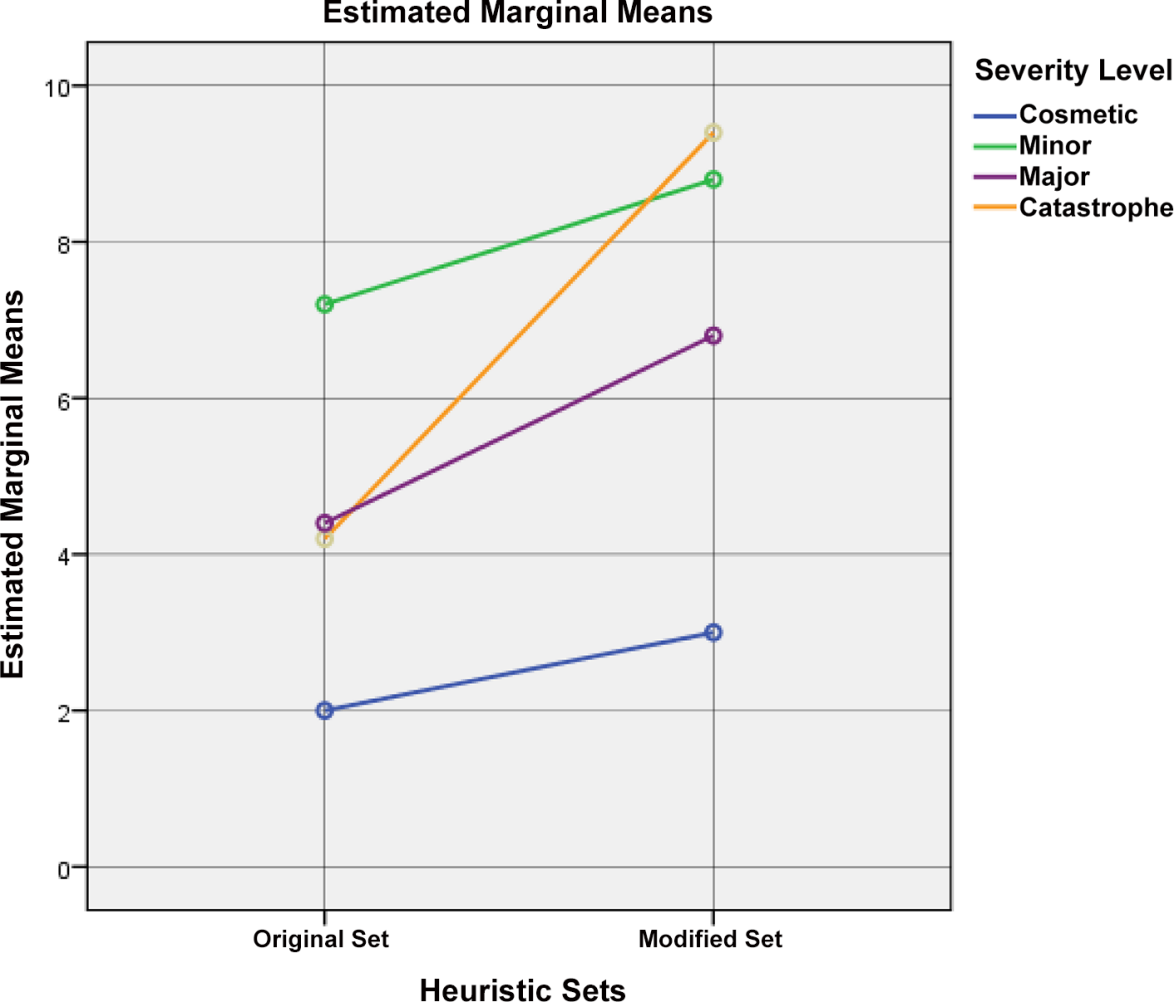
Estimated marginal means of problem identification by both sets of heuristics.

The graph of the profile plot as shown in Fig 8 clearly indicates the effectiveness of problem identification for different severity levels has increased by using the modified set of heuristics. The highest effectiveness of the modified set can be seen in the problem identification of the severity level of major, followed by catastrophe, cosmetic and minor.

H2 –The 5 improved heuristics in the modified set will be able to identify a higher number of usability problems than the corresponding heuristics in the original set: The analysis of SPANOVA results for four groups shows that there is no effect on the variables between *Heuristic Groups and Severity Level* [F(3, 16) = 1.665, p> .05] because of the first 5 heuristics of the modified set. The estimated marginal means show a significant mean difference only between the second 5 heuristics of the original set and the corresponding improved heuristics of the modified set (0.90) (see S6 Table). The SPANOVA results for two groups (group 2 and 4) show the main effect on the variables Severity Level * Heuristic Groups [F(3, 16) = 0.43, p< .05]. Therefore, the null hypothesis is rejected; this means that the improved heuristics of the modified set are significantly effective in the problem identification. The results of these tests indicate that there are significant differences in the problem identification between both systems using the improved heuristics of the modified set and the corresponding heuristics in the original set.

**Fig 9:**
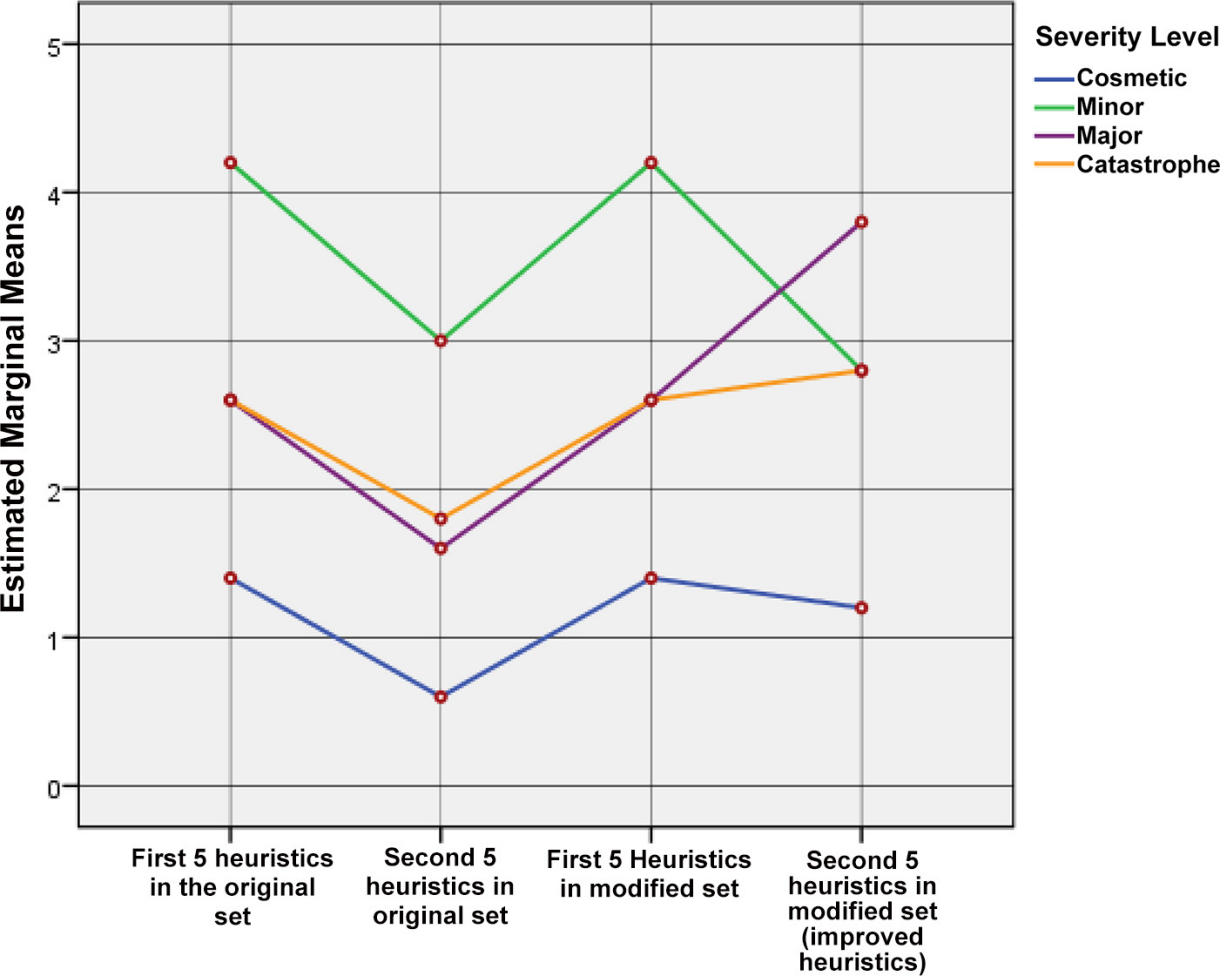
Estimated marginal means of problem identification among four groups at different severity levels.

**Fig 10:**
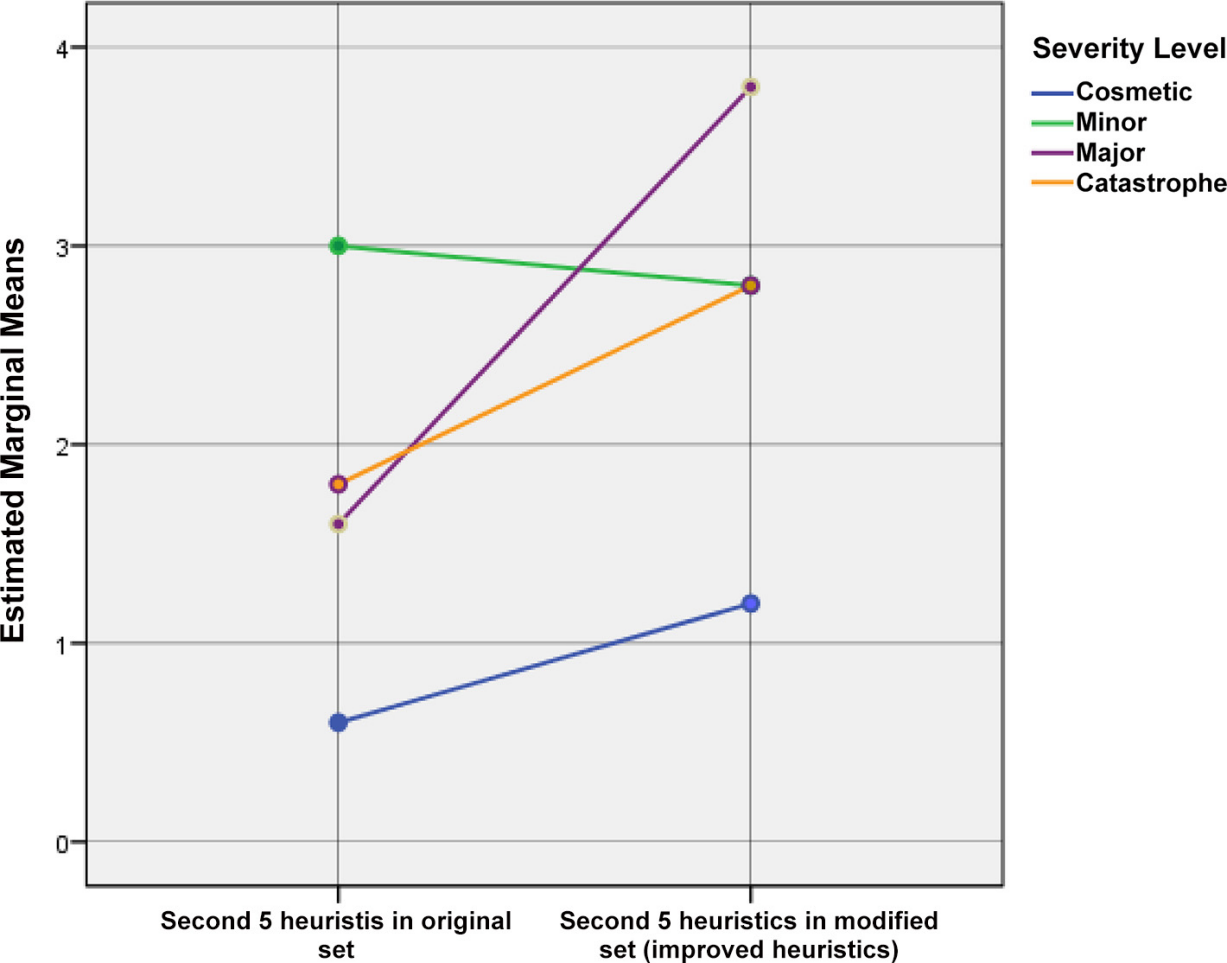
Estimated marginal means of problem identification by groups 2 and 4 at different severity levels.

The graph of the profile plots (Figs 9 and 10) clearly indicates that the effectiveness of problem identification for the different severity levels has increased by using the improved heuristics. The effectiveness of the improved heuristics can be seen in the problem identification of severity type major, followed by catastrophe and cosmetic. Although there is a small decrement in problem identification in the severity type minor, it does not affect the rejection of null-hypothesis. The SPANOVA results show that even with this smallest weakness in the severity type minor, the improved heuristics are still more effective than the corresponding heuristics in the original set.

**Fig 11:**
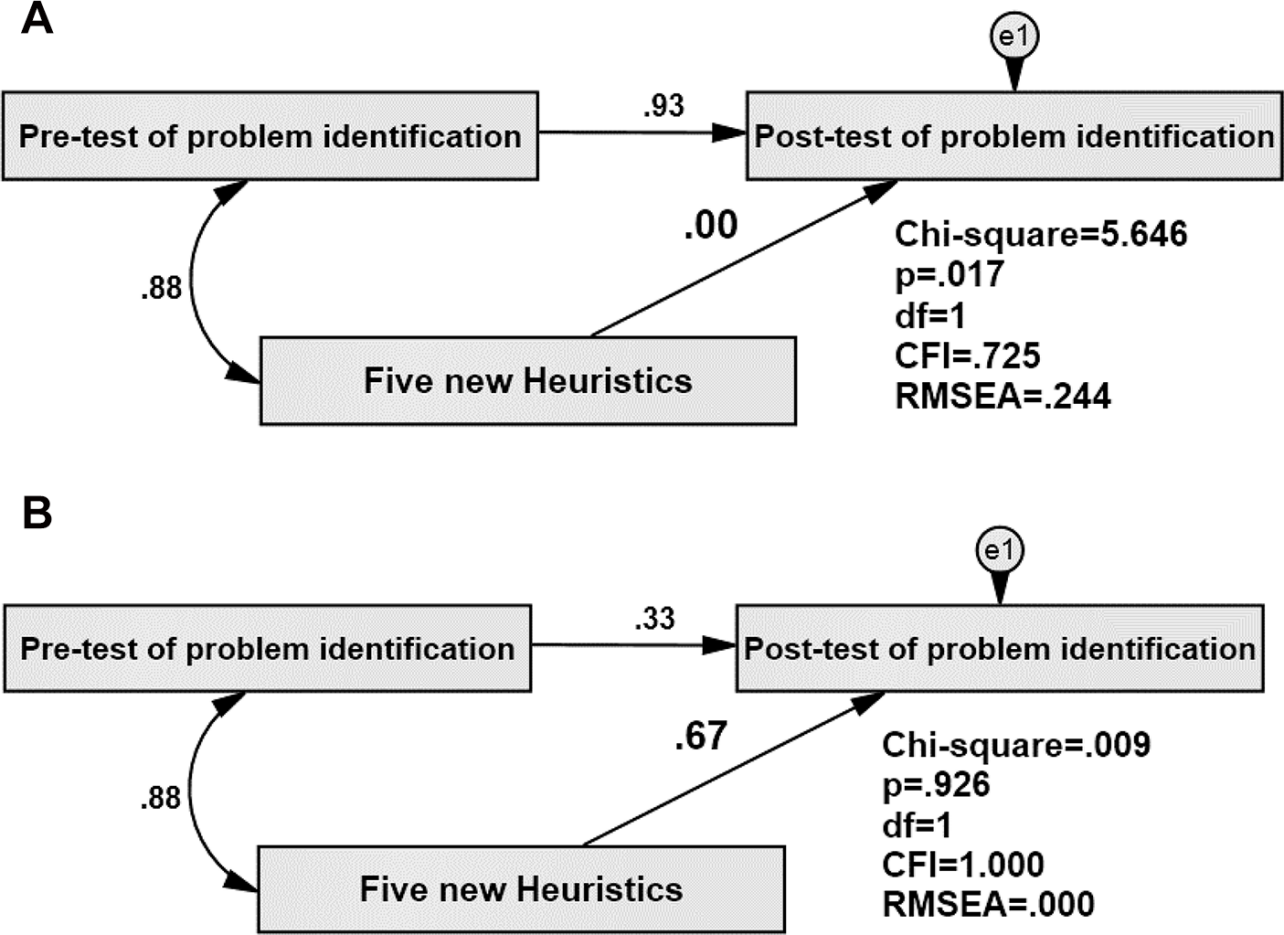
Analysed model when a = 0.

H3 –The 5 new heuristics will contribute to the identification of problems using the modified set: The results of analysing the model when a = 0 is shown in Fig 11. The results show that when a = 0, the model is not fit to collect data and the null hypothesis is rejected.

**Fig 12:**
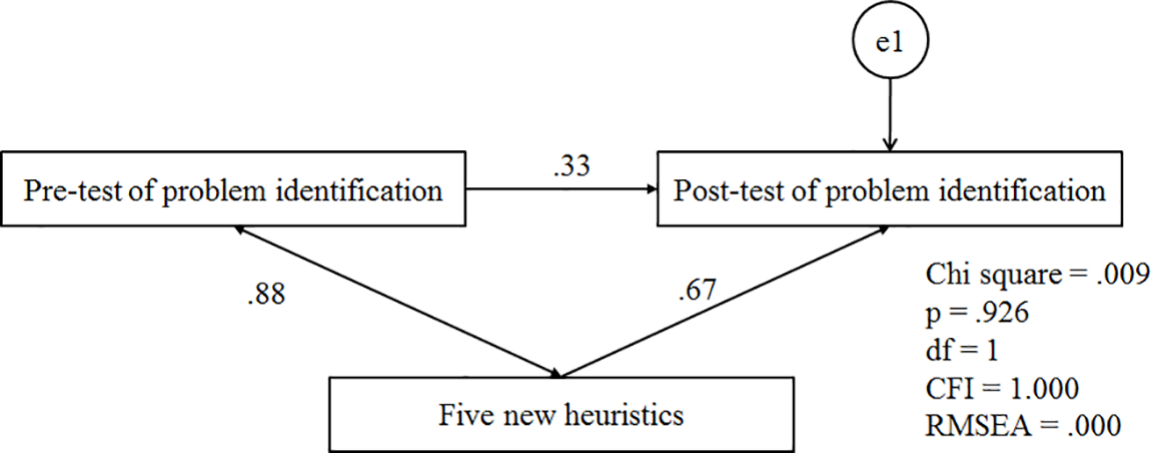
Analysed model when a≠0.

Fig 12 shows the results of analysing the model by considering the effectiveness of the new heuristics. Based on the value of a = .67, P >.05, CFI>.90 and RMSEA < .08, we can conclude that this model is fit to collect data. The value of Chi square in this model is .009, which is smaller than Chi square in Fig 11; that proves the effectiveness of the new heuristics in the problem identification.

The findings indicate that the model which considers the effect of the new heuristics is the correct model fit to collect data. Generally, all the tested indexes such as Chi square (CMIN), CFI and RMSEA show that the model which takes the five new heuristics into account is better than the others.

## Usability Study 2 –Evaluation of an Interactive System Being Developed

This section describes participants involved, instruments used, study protocol, followed by presentation and discussion of results.

### 6.1. Participants and recruitment

The participants’ profiles and the recruitment procedure for this study are similar to those of the first study so as to ensure consistency for both studies. Seven participants were contacted through email, from which three participants gave positive replies and indicated willingness to be part of this study.

### 6.2. Instrument used

A serious game entitled **‘Vocab builder’** was being developed at the multimodal interaction research lab, faculty of computer science and information technology, University of Malaya. The purpose of this game is to facilitate children with ASD in acquiring vocabulary while playing games. A child can select certain level of complexity (easy, medium, hard, or harder) to play and then choose a category of words he or she faces difficulties with. For each category selected, he or she needs to identify the correct object; for instance, if the selected category is ‘bird’, he or she will be shown a jungle scene with different birds flying around, and he or she will be asked to select a specific bird.

### 6.3. Study protocol

Participants received through email a modified set of heuristics and a URL of the serious game. Participants were briefed via Skype before they started their evaluation. This evaluation was conducted in two different phases and the details are described below.

#### 6.3.1. Phase 1

Participants evaluated the serious game for children with ASD and submitted a report of usability problems identified through email. We compiled all the reports from the participants and communicated the compiled list of usability problems to the developers of the serious game. The developers worked on all the problems and fixed them by making the necessary changes to the serious game.

#### 6.3.2. Phase 2

The updated version of the system was uploaded at the same mentioned URL so that the experts could download both copies of the systems for comparison. A copy of report containing the problems found by participants was emailed to them in the form of questionnaire; for each problem, they were asked to specify if the identified problem had been fixed, remained unresolved, or they were unsure about it. The participants returned the completed questionnaire through email after they had performed the evaluation.

### 6.4. Results

The results of both phases of the study are shown in Table 11; it can be seen that in Phase 2, (N = 40 of 43, 93%) of the problems identified in Phase 1 had been fixed, while 2 new problems were detected in the updated serious game. This shows that the modified set of heuristics played a role in the evaluation of the interactive systems during the design and development process to identify and fix usability problems in the systems.

**Table 11:** Results of both phases of study.

Expert	Phase 1	Phase 2			
	Problems found	Problems fixed	Problems unresolved	Not sure	New problems identified
1	15	15	-	-	2
2	16	15	1	-	-
3	12	10	2	-	-
Total	43	40	3	-	2

## Conclusion

In this research, we adapted and improved the original set of heuristics by Nielsen and developed a modified set of 15 heuristics based on literature review on guidelines of interactive systems for children with ASD. When comparing the evaluation results of the modified set with those of the original set in Study 1, the analysis shows the following details:

1. Five new heuristics effectively contributed to the success of the experimental group in identifying problems.
2. Responsiveness heuristic was violated most frequently among the five new heuristics.
3. Five improved heuristics of the modified set helped in finding more usability problems than the corresponding heuristics of Nielsen. There is a significant difference in effectiveness between the five improved heuristics and their corresponding heuristics in the original set.
4. Consistency, minimalist and control heuristics were violated more frequently than others using the 5 same and 5 improved heuristics of the modified set.
5. Overall, the modified set is significantly more effective than the original set.

The analysis of results in Study 2 ascertained the role of the modified set in identifying problems in the systems being developed.

In this research, we compiled a list of guidelines from various sources of literature for the design and development of an interactive system for children with ASD. Designers can incorporate the compiled guidelines in the interactive systems designed for children with ASD and use the modified set of heuristics for the evaluation of the system by experts. Consequently, children with ASD would have less difficulty in using these systems.

The academic staff and the researchers served as evaluators of the interactive systems for children with ASD in this study. However, in future studies, researchers can consider involving HCI practitioners for the evaluation of systems for children with ASD. In addition, more interactive systems, especially the systems being developed can be evaluated to find out the effectiveness of the modified set of heuristics.

## Supporting Information

S1 Table Design guidelines from [29]. (DOCX)

S2 Table Design guidelines from [31]. (DOCX)

S3 Table Design guidelines from [30]. (DOCX)

S4 Table Measure of Agreement (Kappa) for experts’ pairs with their p-value. (DOCX)

S5 Table Intra-class Correlation Coefficient (ICC) results. (DOCX)

S6 Table Estimated marginal means between severity level and heuristics groups. (DOCX)
