# Correction: Heuristics to Evaluate Interactive Systems for Children with Autism Spectrum Disorder (ASD)

**DOI:** 10.1371/journal.pone.0136977

**Published:** 2015-08-24

**Authors:** Kamran Khowaja, Siti Salwah Salim

Dr. Adeleh Asemi should be included in the author byline. She should be listed as the third author, and her affiliation is 1: Faculty of Computer Science and Information Technology, University of Malaya, 50603, Kuala Lumpur, Malaysia. The contributions of this author are as follows: Analyzed the data, contributed reagents/materials/analysis tools, and wrote the manuscript.

The correct citation is: Khowaja K, Salim SS, Asemi A (2015) Heuristics to Evaluate Interactive Systems for Children with Autism Spectrum Disorder (ASD). PLoS ONE 10(7): e0132187. doi:10.1371/journal.pone.0132187

The Corresponding Author may be contacted at kamran.khowaja@gmail.com in addition to the published email address, kamran.khowaja@siswa.um.edu.my.

There is an error in Fig 4. Please see the corrected Fig 4 here.

**Fig 4:**
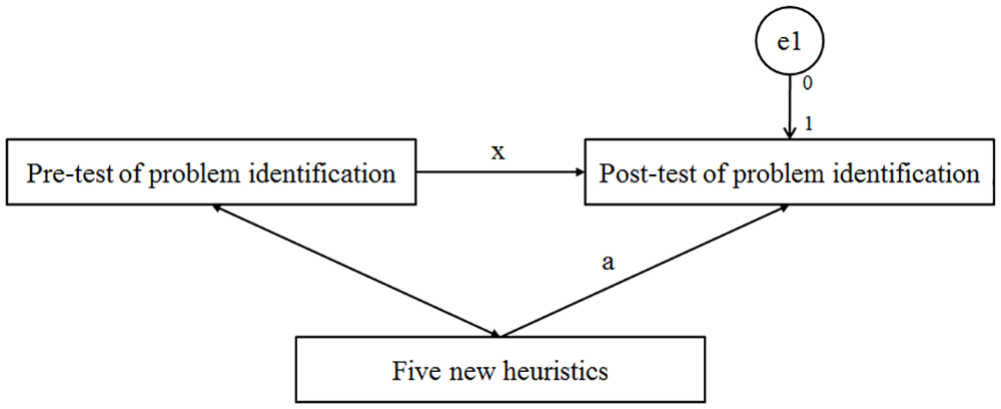
Hypothesis model.

Fig 11 is incorrect. Please see the correct Fig 11 here.

**Fig 11:**
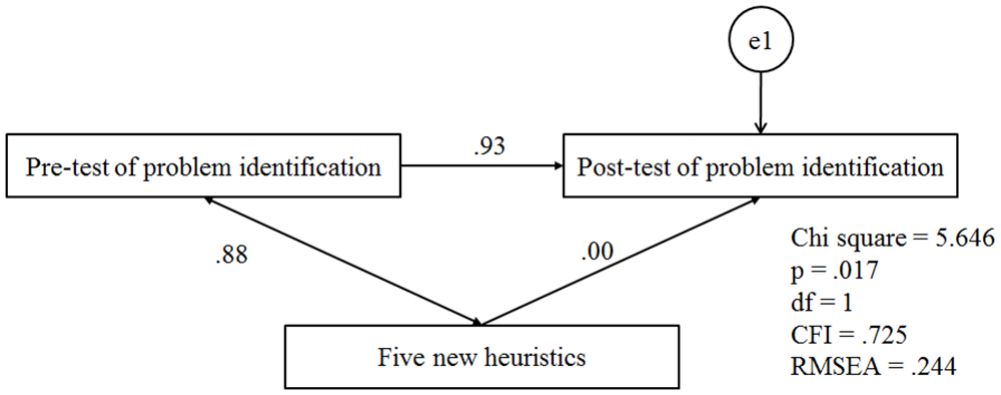
Analysed model when a = 0.
