# Supplementary material for: Heuristics to Evaluate Interactive Systems for Children with Autism Spectrum Disorder (ASD)
Source: PLoS One. 2015 Jul 21;10(7):e0132187. doi: 10.1371/journal.pone.0132187 (PMC4510389; doi:10.1371/journal.pone.0132187)
Supplement: S1 Table — (DOCX) [file pone.0132187.s001.docx]

*S1 Table. Design guidelines from [29]*

| 1. The individual student should be considered at all times as the layout and design of the software progresses. Does the student learn best visually, auditorily? Should reinforcement be visual or auditory? How much stimulation is too much/not enough?  2. The student's motivational level should dictate what needs to be incorporated into the software to increase the student's response level.  3. The student's response should be directly related to a request for responding in the software. The relationship between his or her behavior and the consequence on the computer screen should be clear to the student.  4. The student should see immediately the consequence of his or her response.  5. The software should be designed to increase the student's opportunity for independent responding and provide reinforcement accordingly.  6. The software should afford the student the opportunity to work with a computer buddy when appropriate.  7. The software should be portable so that it can be used at home or in the general education classroom.  8. Digitized speech, not synthetic speech, should be used.  9. The software should speak directions to the student in a clear and direct manner.  10. Voices of people with whom the student interacts on a regular basis should be used, with familiar voices fading to new and unfamiliar voices.  11. A variety of voice intonations should be used.  12. The goal here is that the student has a repeated opportunity to associate the positive outcomes available from responding to the verbal instructions in the software. If the student does not respond to the instructions, nothing happens.  13. Pictures, stimulus materials, or instructional examples used in teacher-directed instruction should be used in the software.  14. Pictures, stimulus materials, or instructional examples found in the age-appropriate general education classroom should be used in the software.  15. Age-appropriate materials found in the natural environment of the student should be used in the software.  16. Opportunities for repeated practice should be built into the software. This will vary depending on the student.  17. The presentation of the skill in the software should be varied. Although some predictability is important, too much predictability may lead to over selectivity on the part of the student.  18. Photographs of real objects rather than drawings (e.g., a photograph of a dog rather than a drawing of a dog) should be used.  19. Voices of significant people in the student's life should be used in the design of the software.  20. Music the student enjoys should be included.  21. Allow the student to take the software home or use it in the general education classroom.  22. The software should support the learning occurring in teacher­directed activities.  23. The software should support the learning occurring at home, in the general education environment, or when playing with peers.  24. The use of multiple, natural examples that occur in the student’s environment can be incorporated into the software design.  25. When the student responds incorrectly to the software, something should still occur (e.g., a sad face appears or a voice says, ‘No, that was not a dog.’).  26. Items included in the software lessons should be chosen only after the student has demonstrated sustained attention to the object or item.  27. Items included in the software lessons should reflect items about which the student has intentionally communicated.  28. New tasks or information should build on previously learned tasks or information.  29. Tasks should engage the child at more than a ‘play’ level.  30. Any computerized lesson should have a real-world counterpart.  31. Mastered task items should be incorporated into the design of the software (e.g., maintenance tasks are interspersed in new learning tasks).  32. Presentation of tasks in the software should be varied so the student has the opportunity to work on previously mastered tasks following work on items he or she is just beginning to learn.  33. Prompts that involve the exaggeration of components of the complex stimulus should be used.  34. The number of cues should be gradually increased. Reinforcement should occur only when the student responds to the task on the basis of multiple cues.  35. Reinforcers used in the design of the software should be varied.  36. Reinforcers that are selected for use in the software should be ‘Student driven,’ that is, keyed to student interests.  37. Reinforcers should be easily changed in the software. Reinforcers should be changed as the student’s interest’s change.  38. Lessons should be designed around discriminations based on size, shape, and/or color.  39. Lessons should make use of multiple components throughout.  40. Once a student has been taught to respond to two cues, systematically teach three cues, four cues, etc.  41. The software should provide prompts if the student doesn't respond within a set time period (e.g., the correct answer flashes, other choices disappear from the screen, other choices fade on the screen, the correct answer is circled or under­ lined).  42. The prompts should be varied so that the student does not anticipate or learn the prompt.  43. As the student gains experience with the software, the prompts should be faded.  44. The software should collect data on how long a student worked on an individual screen, the number of correct responses, the number of incorrect responses, etc. |
| --- |
