# Supplementary material for: Heuristics to Evaluate Interactive Systems for Children with Autism Spectrum Disorder (ASD)
Source: PLoS One. 2015 Jul 21;10(7):e0132187. doi: 10.1371/journal.pone.0132187 (PMC4510389; doi:10.1371/journal.pone.0132187)
Supplement: S2 Table — (DOCX) [file pone.0132187.s002.docx]

*S2 Table. Design guidelines from [*[*31*](#_ENREF_31)*]*

| 45. Visual cues: children with autism should be provided visual cues wherever needed. This will help them to better understand what they are supposed to do [[47](#_ENREF_47),[48](#_ENREF_48)].  46. Repetition: children with autism shall be given facility to repeat activity as many times as they want [[47](#_ENREF_47),[49](#_ENREF_49),[50](#_ENREF_50)].  47. Structure: Children with autism thrive in a structured environment.  Establish a routine and keep it as consistent as possible.  In a world that’s ever changing, routine and structure provide great comfort to a child on the autism [[47](#_ENREF_47),[51](#_ENREF_51)].  48. Predictability: children with autism are likely to prefer interface which makes it easier for them to predict and interact with system [[47](#_ENREF_47),[49](#_ENREF_49)].  49. Controllability: children with autism tend to work on interface which they can control [[47](#_ENREF_47),[49](#_ENREF_49)].  50. Interest: children with autism prefer to use and work the object which are of their interest. The system should allow them to make a selection of their own choice [[47](#_ENREF_47),[49](#_ENREF_49)].  51. Unhurried pace: children with autism should be allowed to spend as much time on the activity as they want. The unrestricted time will help them to improve their learning [[47](#_ENREF_47),[50](#_ENREF_50)].  52. Children with autism do not like change, consistency needs to be applied throughout the interface as to not deter the user. Maintaining this strict rule means the user can perform other tasks with ease such as navigation.  53. Visual learning is most preferred for children with autism especially at a young age. The use of familiar icons can aid the child in learning and following the system with relative ease. Imagery could have a positive influence within a learning application. The reason being is that ‘…few demands on the child's cognitive, linguistic and memory skills'. Simply put, imagery is more interactive and fun for the child therefore they have less hard of a time trying to remember tasks, and can process ideas more easily.  54. Location indicators are an effective way of letting the user knows their exact location within the system. Such indicators could be ‘breadcrumb' navigation techniques meaning that they are made aware of where they have come from and where they are. If properly implemented into a system, this technique can aid in preventing the child becoming agitated if they get lost. If this navigation is not consistently on each page then this results in a change in the child's routine which may cause the child to be upset, which in turn can reduce enjoyment of the application and may prevent users from gaining information.  55. Specific font styles e.g. sizes should be implemented to aid in the representation of information for the user, along with headings of or new sections within the application. The child must also be able to read the text provided to them and that its presentation is important in order to aid the child in a fuller learning experience.  56. An interface agent can provide descriptions of tasks and controls within the application so make the user at ease from the outset. Again, like the lack of location indicators, could cause agitation and frustration f or the child.  57. Animations are a great tool for easing a child into an activity. It provides a break from the norm with the use of character animation instead of text based learning. Characters on screen should not be distracting to the user but bring the text on the screen to life. A good application will be effective in making the child want to use it rather than being asked to by the teacher.  58. Text-based interfaces were not as efficient as those with visual aids and graphical metaphors. This reiterates the effectiveness for an onscreen character.  59. Children including those with autism require feedback immediately, as the lack of result will deter the user.  60. If they press a button and nothing happens, the child will immediately try to press the button again and again until a result occurs. Constant audio such as voice over or sound effects can be annoying for older users yet younger children expect it. This is a good point to mention, as if an older child were to use an application with constant audio and imagery, they may leave. Each age group is different to one another.  61. Recommended screen are of 760px * 410px minimum to provide enough room for the user to maneuver around the application with relative ease. This screen size will prevent or significantly reduce the need to scroll to view new information.  62. Minimum button size of 27px * 27px should be implemented for those without learning difficulties. However since this application is for users with autism, the buttons need to be significantly bigger so that they can be pressed more easily, whether it is by finger on a touch screen or by clicking a mouse. |
| --- |
