# Supplementary material for: Heuristics to Evaluate Interactive Systems for Children with Autism Spectrum Disorder (ASD)
Source: PLoS One. 2015 Jul 21;10(7):e0132187. doi: 10.1371/journal.pone.0132187 (PMC4510389; doi:10.1371/journal.pone.0132187)
Supplement: S3 Table — (DOCX) [file pone.0132187.s003.docx]

*S3 Table. Design guidelines from [*[*30*](#_ENREF_30)*]*

| 63. Children with autism are likely to prefer predictable, structured and controlled procedures and environments and, possibly consequently, they like inanimate objects, machines and computers.  64. Children with autism are generally thought to be highly visual thinkers and learners.  65. Children with autism might have little apparent understanding of joint attention or of shared points of reference such as references made to remote objects by pointing. Children with autism are not incapable of such behaviors. However an interface designer should not rely on a child with autism understanding a reference made to an object by pointing at it.  66. Children with autism may not be able to use a standard keyboard or mouse.  67. Children with autism may be highly sensitive to noise, finding intolerable noise which is barely perceptible or unremarkable to others.  68. Children with autism generally enjoy repetition and may engage in repetitive activity to the detriment of other activities.  69. Children with autism have a tendency to focus on particular details, that is, they tend to employ local rather than global integration. A preference for local integration was shown in children with autism in the case of voluntary selective attention, that is, when the participants are not given guidance on what to attend to. Again, children with autism are not incapable of focusing on the global picture rather than on detailed aspects; children with autism could attend to the whole picture rather than it‘s parts if they were overtly primed to attend to the global level. However, interface designers should be aware that children with autism may focus on seemingly irrelevant detail.  70. Children with autism may find failure very debilitating, as they might be employing strategies which worked in the past |
| --- |
