# Supplementary material for: Heuristics to Evaluate Interactive Systems for Children with Autism Spectrum Disorder (ASD)
Source: PLoS One. 2015 Jul 21;10(7):e0132187. doi: 10.1371/journal.pone.0132187 (PMC4510389; doi:10.1371/journal.pone.0132187)
Supplement: S4 Table — (DOCX) [file pone.0132187.s004.docx]

S4 Table. Measure of Agreement (Kappa) for experts’ pairs with their p-value

|  | **E1** | **E2** | **E3** | **E4** | **E5** | **E6** | **E7** | **E8** | **Kappa** |
| --- | --- | --- | --- | --- | --- | --- | --- | --- | --- |
| **E1** | 1.000  (P=.000) | .656  (P=.000) | .483  (P=.000) | .224  (P=.05) | .372  (P=.003) | .489  (P=.000) | .477  (P=.001) | .323  (P=.000) | 0.432 |
| **E2** | .656  (P=.000) | 1.000  (P=.000) | .659  (P=.000) | .318  (P=.004) | .486  (P=.000) | .323  (P=.000) | .656  (P=.000) | .494  (P=.000) | 0.513 |
| **E3** | .483  (P=.000) | .659  (P=.000) | 1.000  (P=.000) | .224  (P=.05) | .375  (P=.01) | .224  (P=.05) | .483  (P=.000) | .318  (P=.004) | 0.395 |
| **E4** | .224  (P=.05) | .318  (P=.004) | .224  (P=.05) | 1.000  (P=.000) | .375  (P=.001) | .310  (P=.000) | .224  (P=.05) | .659  (P=.000) | 0.333 |
| **E5** | .372  (P=.003) | .486  (P=.000) | .375  (P=.01) | .375  (P=.001) | 1.000  (P=.000) | .171  (P=.093) | .372  (P=.003) | .233  (P=.009) | 0.341 |
| **E6** | .489  (P=.000) | .323  (P=.000) | .224  (P=.05) | .310  (P=.000) | .171  (P=.093) | 1.000  (P=.000) | .233  (P=.001) | .656  (P=.000) | 0.344 |
| **E7** | .477  (P=.001) | .656  (P=.000) | .483  (P=.000) | .224  (P=.05) | .372  (P=.003) | .233  (P=.001) | 1.000  (P=.000) | .323  (P=.000) | 0.395 |
| **E8** | .323  (P=.000) | .494  (P=.000) | .318  (P=.004) | .659  (P=.000) | .233  (P=.009) | .656  (P=.000) | .323  (P=.000) | 1.000  (P=.000) | 0.429 |
