# Supplementary material for: Heuristics to Evaluate Interactive Systems for Children with Autism Spectrum Disorder (ASD)
Source: PLoS One. 2015 Jul 21;10(7):e0132187. doi: 10.1371/journal.pone.0132187 (PMC4510389; doi:10.1371/journal.pone.0132187)
Supplement: S5 Table — (DOCX) [file pone.0132187.s005.docx]

S5 Table. Intra-class Correlation Coefficient (ICC) results

|  | **Intra-class Correlation** | **95% Confidence Interval** | | **F Test with True Value 0** | | | |
| --- | --- | --- | --- | --- | --- | --- | --- |
|  |  | **Lower Bound** | **Upper Bound** | **Value** | **df1** | **df2** | **Sig** |
| Single Measures | .262 | .024 | .863 | 4.550 | 3 | 27 | .010 |
| Average Measures | .780 | .198 | .984 | 4.550 | 3 | 27 | .010 |
