# Supplementary material for: Heuristics to Evaluate Interactive Systems for Children with Autism Spectrum Disorder (ASD)
Source: PLoS One. 2015 Jul 21;10(7):e0132187. doi: 10.1371/journal.pone.0132187 (PMC4510389; doi:10.1371/journal.pone.0132187)
Supplement: S6 Table — (DOCX) [file pone.0132187.s006.docx]

S6 Table. Estimated marginal means between severity level and heuristics groups

| **Severity Level** | ***Heuristics* *Groups*** | **Mean** | **Std. Error** | **95% Confidence Interval** | |
| --- | --- | --- | --- | --- | --- |
|  |  |  |  | **Lower Bound** | **Upper Bound** |
| Cosmetic | 1 | 1.400 | 1.142 | -1.022 | 3.822 |
|  | **2** | **.600** | 1.032 | -1.588 | 2.788 |
|  | 3 | 1.400 | 1.142 | -1.022 | 3.822 |
|  | **4** | **1.200** | 1.056 | -1.038 | 3.438 |
| Minor | 1 | 4.200 | 1.142 | 1.778 | 6.622 |
|  | **2** | **3.000** | 1.032 | .812 | 5.188 |
|  | 3 | 4.200 | 1.142 | 1.778 | 6.622 |
|  | **4** | **2.800** | 1.056 | .562 | 5.038 |
| Major | 1 | 2.600 | 1.142 | .178 | 5.022 |
|  | **2** | **1.600** | 1.032 | -.588 | 3.788 |
|  | 3 | 2.600 | 1.142 | .178 | 5.022 |
|  | **4** | **3.800** | 1.056 | 1.562 | 6.038 |
| Catastrophe | 1 | 2.600 | 1.142 | .178 | 5.022 |
|  | **2** | **1.800** | 1.032 | -.388 | 3.988 |
|  | 3 | 2.600 | 1.142 | .178 | 5.022 |
|  | **4** | **2.800** | 1.056 | .562 | 5.038 |
